# Population Structure Analysis and Candidate Gene Screening for Twinning Trait in Simmental Cattle

**DOI:** 10.3390/ani16101567

**Published:** 2026-05-21

**Authors:** Kailun Ma, Xiaoyun Liang, Lei Xu, Xue Li, Hongkun Zhao, Jiajie Huang, Jingjing Wen, Menghua Zhang, Dan Wang, Xixia Huang, Qiuming Chen

**Affiliations:** College of Animal Science, Xinjiang Agricultural University, Urumqi 830052, China; makailun0829@163.com (K.M.); 15823168822@163.com (X.L.); q609468041@sina.com (L.X.); lixueli1126@163.com (X.L.); zhaohongkun4416@163.com (H.Z.); jayjayh@yeah.net (J.H.); w18293472033@126.com (J.W.); zhangmenghua810@126.com (M.Z.); wangdan01100330@163.com (D.W.); au-huangxixia@163.com (X.H.)

**Keywords:** Simmental cattle, population structure, twinning trait, selection signature, candidate genes

## Abstract

Chinese Simmental cattle are a high-quality breed developed through long-term crossbreeding and selection after their introduction into China and have become the main dual-purpose cattle population in the Xinjiang region. To deeply dissect the population structure, characteristics of the population structure, and the genetic basis of the twinning trait, this study focused on Xinjiang Chinese Simmental cattle as the main research subject. It integrated genomic data from global public databases to systematically conduct population structure analysis, genetic relationship analysis, and genome-wide selection signature analysis. Population genetic analysis revealed that the IBS matrix and G matrix indicated that some individuals from different geographical origins exhibited distant genetic relationships; the Xinjiang population showed the fastest LD decay, suggesting abundant genetic diversity; the inbreeding coefficient based on Runs of Homozygosity (ROH) across populations ranged from 0.036 to 0.063; principal component analysis and phylogenetic tree showed that some individuals from different geographical origins had certain genetic interconnections; admixture analysis indicated that K = 5 was the optimal model, with each population exhibiting clear genetic differentiation and admixture characteristics. Furthermore, by combining *F*_st_ and θπ analysis (comparing the Xinjiang population with other geographical populations), a total of 89 candidate genes associated with the twinning trait in Xinjiang Chinese Simmental cattle were screened, including *CYP19A1*, *HORMAD1*, *GRB14*, *CADM2*, *CXCR4*, and others that have been reported to be closely related to oogenesis and reproductive function. In summary, this study explores genome-wide genetic differences among Simmental cattle populations from different regions, deepens our understanding of their population structures, and offers new candidate genes and molecular markers for high-fecundity breeding in Simmental cattle.

## Simple Summary

To dissect the population structure and the genetic basis of the twinning trait in Chinese Simmental cattle, this study focused on populations from the Xinjiang region and integrated global genomic data to conduct analyses of population genetic structure and selection signatures. We found that individuals from different geographic origins exhibited relatively close genetic relationships, and we assessed the population’s inbreeding level. Simmental cattle in the Xinjiang region exhibited abundant genetic diversity, and certain genetic connections existed among populations of different geographic origins. In the Chinese Simmental population, some individuals consisted of a single ancestral component, whereas the majority were composed of two or three ancestral components. Through selection signature analysis, 89 candidate genes associated with the twinning trait were identified, including *CYP19A1*, *HORMAD1*, *GRB14*, *CADM2*, *CXCR4*, and others. These findings provide an important basis for revealing the genetic background of Xinjiang Chinese Simmental cattle and for molecular breeding of high fecundity.

## 1. Introduction

Simmental cattle (also known as Fleckvieh) originated in the Alpine region of Switzerland and emerged as a breed in Central Europe in the 1830s. They are a famous dual-purpose breed with red-and-white patches, featuring a fast growth rate, excellent meat and milk production, and strong adaptability [1]. Today, this breed is found in more than 120 countries and regions, with a global population exceeding 50 million, ranking second only to Holstein. It is mainly distributed in France, Austria, Germany, and Switzerland [2,3], and has become one of the core breeds in China’s cattle industry.

In recent years, with the rapid development of high-throughput sequencing technologies and bioinformatics methods, population genetics research has advanced from traditional phenotypic observation to genomic analysis. Traditional studies rely on phenotypic traits and pedigree records, which have limitations such as insufficient genetic information coverage and low resolution [4]; in contrast, genome-wide analysis technologies enable researchers to perform multi-dimensional, high-precision analysis of the genetic structure of cattle populations. Among these, selection signature analysis, as a core technique for identifying genomic regions under selection and mining selected genes associated with economic traits, can detect genomic differentiation between populations and screen for selection regions left by long-term artificial breeding, thereby providing an effective approach for understanding the genetic mechanisms underlying complex traits.

The twinning trait is an important reproductive economic trait in Simmental cattle, and its genetic improvement is of great significance for enhancing herd reproductive efficiency and reducing production costs. The twinning trait is a typical quantitative trait characterized by low heritability and complex phenotypic expression, influenced by multiple genes and environmental factors [5]. Traditional phenotypic selection has achieved slow genetic progress, and there is an urgent need to dissect its genetic basis and identify candidate genes through molecular genetic approaches, thereby providing theoretical support for marker-assisted breeding. Selection signature analysis offers a feasible approach for localizing selection regions associated with the twinning trait. Genome-wide association studies (GWAS) have identified multiple genes and variants associated with the twinning trait in cattle, involving biological processes such as reproductive hormone secretion, follicular development, and embryo implantation. For example, Lett et al. [6] identified 12 genomic regions (BTA3, BTA6, BTA18) associated with twinning rate in North American Holstein cattle and screened out three candidate genes: *CCDC141*, *GABRG3*, and *ESR1*. Widmer et al. [7] mapped a QTL covering *LHCGR* and *FSHR* on BTA11 in Swiss and German Holstein cattle. Moioli et al. [8] performed a GWAS based on 54K SNP data on 1200 Italian Maremmana cows, identified three regions (BTA6, BTA14, BTA19) significantly associated with twinning rate, and detected a significant SNP on BTA24. Although GWAS has revealed several genomic regions associated with reproductive traits [9], studies using selection signature analysis to screen for genes related to the twinning trait remain relatively limited.

This study focuses on the Chinese Simmental cattle population in the Xinjiang region as the main research subject, integrates global Simmental cattle genomic data from public databases, and uses whole-genome resequencing technology to obtain high-density SNP markers. It evaluates genomic genetic diversity, the genetic relationship matrix, and individual genetic distances to reveal population structure and genetic relationships. Furthermore, the combined analysis of the population differentiation index (*F*_st_) and the nucleotide diversity ratio (θπ ratio) is employed to detect genome-wide selection signatures in Xinjiang Chinese Simmental cattle and to screen candidate genes potentially associated with the twinning trait, to provide a theoretical basis for the genetic resource conservation and precision breeding of Chinese Simmental cattle in the Xinjiang region.

## 2. Materials and Methods

### 2.1. Materials Source

In this study, a total of 77 healthy Simmental cows were selected for genomic analysis, including 57 individuals from Xinjiang Yili Chuangjin Benniu Animal Husbandry Co., Ltd. (Yili, China), and 20 individuals from the Third Pasture Farm of Xinjiang Hutubi Breeding Cattle Farm Co., Ltd. (Hutubi, China) Blood samples (10 mL per cow) were collected from the caudal vein into EDTA-containing tubes, stored at −20 °C, and subsequently transported to the laboratory for genomic DNA extraction.

To investigate the population genetic structure of Simmental cattle from different geographical origins, this study further incorporated, in addition to the 77 newly collected samples, the resequencing data of 79 Simmental individuals previously sequenced in our laboratory from Xinjiang Yili Chuangjin Benniu Animal Husbandry Co., Ltd. (Yili, China), and simultaneously downloaded genome resequencing data of Simmental cattle and Huaxi cattle from worldwide sources from the NCBI database (https://www.ncbi.nlm.nih.gov/, accessed on 1 March 2026). After stringent quality control (QC) of the downloaded raw resequencing data, low-quality data with a sequencing depth below 8× were removed, ultimately yielding 206 qualified resequencing data entries. These 206 qualified resequencing entries originated from four countries, among which the geographical origins of 94 Simmental cattle were clearly documented, with the specific distribution as follows: 26 from Gansu Province, China; 18 from Inner Mongolia Autonomous Region, China; 17 from the United States; 31 from Canada; and 2 from Germany. The specific origins of the remaining 27 Simmental cattle were not clearly recorded. In addition, all 85 Huaxi cattle originated from the Inner Mongolia region of China. All the above samples (77 newly collected, 79 previously sequenced, and 206 downloaded from NCBI) were merged into eight populations based on geographical origin: Xinjiang, Gansu, Inner Mongolia, the United States, Canada, Germany, unknown-origin Simmental, and Inner Mongolia Huaxi, for subsequent genomic data description, SNP detection, and annotation. To identify selection signatures associated with the twinning trait, this study used calving records from 156 Chinese Simmental cattle in the Xinjiang region. Among them, 57 individuals with twin-calving records were assigned as the experimental group, while 21 individuals with no twin-calving record and with at least four parities were assigned as the control group for subsequent selection signature analysis. The remaining individuals, together with the NCBI public data, were divided by geographical origin into six control populations (Xinjiang, Gansu, Inner Mongolia, US Simmental, Canadian Simmental, and Inner Mongolia Huaxi) for subsequent allele frequency comparisons (Supplementary Table S1).

### 2.2. DNA Extraction and Sequencing

DNA was extracted from Simmental cattle blood samples using the standard phenol-chloroform method. The purity, integrity, and concentration of the extracted DNA were assessed using agarose gel electrophoresis and a Nanodrop 2000 ultra-micro spectrophotometer (Thermo Fisher Scientific, Waltham, MA, USA), and DNA concentration was further quantified using a Qubit 2.0 fluorometer (Thermo Fisher Scientific, USA). The DNA samples were then stored at −80 °C. DNA samples that passed quality control were shipped on dry ice to Shijiazhuang Borui Di Biotechnology Co., Ltd. (Shijiazhuang, China) for whole-genome resequencing. The library insert size was 350 bp, with a paired-end read length of 150 bp, and sequencing was performed on the DNBSEQ-T7 platform. The target sequencing depths for the newly collected samples, the previously sequenced samples from our laboratory, and the public data were 10×, 40×, and 10×, respectively.

### 2.3. Sequencing Data Quality Control and Alignment

First, FastQC (https://www.bioinformatics.babraham.ac.uk/projects/fastqc/, accessed on 20 March 2026) was used to perform a comprehensive quality assessment of the raw sequencing data. Subsequently, fastp v0.23.4 software [10] was employed to conduct stringent quality control on the raw data files (fastq format) generated from paired-end sequencing. Parameters were set as ‘fastp -i -I -o -O -w 4 -q 20 -n 2 -u 30’ to remove low-quality reads and adapter sequences, thereby obtaining clean reads for subsequent data analysis. Based on the BWA-MEM algorithm of the BWA v0.7.17 software [11], the high-quality clean reads obtained after quality control were aligned to the bovine reference genome (ARS-UCD1.2). Then, the SortSam and MarkDuplicates modules of Picard v2.25.5 were used to process the sequencing data, removing PCR duplicates to reduce interference, and the high-quality alignment results were used for downstream analysis.

### 2.4. SNP Detection

To detect and filter raw SNPs, the HaplotypeCaller, CombineGVCFs, GenotypeGVCFs, SelectVariants, and VariantFiltration modules of GATK v4.4.0.0 software [12] were used, with the following quality control criteria: QD < 2.0, FS > 60.0, SOR > 3.0, MQ < 40.0, MQRankSum < −12.5, QUAL < 30.0, ReadPosRankSum < −8.0. Subsequently, the MergeVcfs utility of GATK was used to merge the VCF files of all chromosomes into a genome-wide VCF file. SNP filtering followed these retention criteria: (1) minor allele frequency (MAF) ≥ 0.05; (2) maximum missing rate ≤ 0.20; (3) Hardy-Weinberg equilibrium test *p*-value > 1 × 10^−6^; (4) quality score (QUAL) ≥ 30; (5) genotype quality (GQ) ≥ 10; (6) only biallelic sites retained. Next, VCFtools v0.1.17 software [13] was used to convert the VCF file into PLINK v1.90 format, and further filtering was applied to remove sites with a missing rate exceeding 5% and individuals with a missing rate exceeding 10%. Finally, the filtered SNPs were compared with the reference genome, and ANNOVAR software v2016-02-01 [14] was used to annotate the SNP loci.

### 2.5. Statistical Analysis

#### 2.5.1. Genetic Distance Matrix and Genetic Relationship Matrix

To evaluate genetic relationships among individuals within the population, PLINK v1.90 was first used to calculate genetic distances for all individuals, and identity-by-state (IBS) genetic distance was employed for individual-level genetic relationship analysis. Meanwhile, GCTA v1.94.1 software [15] was used to construct the genetic relationship G-matrix among all Simmental individuals. Finally, the IBS genetic distance matrix and the genetic relationship G-matrix were visualized as heatmaps in R v4.4.1 to display the patterns of genetic relationships among individuals intuitively.

#### 2.5.2. ROH Analysis

PLINK v1.90 software was used to detect runs of homozygosity (ROH). The sliding window method was applied to autosomes with the following parameters: ‘--homozyg-density 50 --homozyg-gap 100 --homozyg-snp 50 --homozyg-window-het 3 --homozyg-window-missing 5 --homozyg-kb 500 --homozyg-window-snp 50 --homozyg-window-threshold 0.05’. The number of ROH in the Simmental cattle population was statistically analyzed. The formula for calculating the inbreeding coefficient using ROH is as follows:

$$F_{\mathrm{ROH}\;}=\;\frac{\sum L_{\mathrm{ROH}}}{L_{\mathrm{genome}}}$$

In the formula, $F_{ROH}$ is the genomic inbreeding coefficient of an individual; $\sum L_{ROH}$ is the total length of all ROH segments in the individual’s genome; $L_{genome}$ is the total physical length of the autosomal genome (the total length of autosomes is approximately 2489.39 Mb). Finally, Origin 2024 software was used for visualization.

#### 2.5.3. Linkage Disequilibrium Analysis

Linkage disequilibrium (LD) analysis was performed on genomic variation data from Simmental cattle of different geographic origins. LD decay was calculated using PopLDdecay v3.42 software [16] with the following parameters: a maximum physical distance of 500 kb was set to calculate the r^2^ values between markers within each population; based on the LD relationships between SNP loci on each chromosome, the average r^2^ decay trend at different distances was statistically analyzed; the LD decay results were visualized using the accompanying Perl script to generate LD decay curves, and the LD decay rate and genomic linkage characteristics of different populations were evaluated.

#### 2.5.4. Population Genetic Structure 

The principal component analysis (PCA) results were visualized using the R package ggplot2. PLINK v1.90 software [17] was used to analyze the genetic relationships among Simmental cattle populations and to construct a genetic distance matrix. The genetic distance matrix file was converted to a format compatible with MEGA v7.0 [18] using a custom Perl script, and a phylogenetic tree was constructed using the neighbor-joining (NJ) method. Finally, the phylogenetic tree was optimized for visualization using the iTOL online tool (https://itol.embl.de/, accessed on 2 April 2026). Population structure analysis was performed using Admixture v1.3.0 software [19], which is simple to operate and fast in computation. Based on the quality-controlled SNP markers and after filtering for linkage information using PLINK v1.90 software, we analyzed individual admixture proportions, population genetic structure, and the extent of gene flow among populations. Considering the diversity of geographic origins of the populations in this study, the number of subpopulations (K) was set from 2 to 10. The cross-validation (CV) error was calculated for each K value to determine the optimal number of ancestral populations, and the result with the lowest CV error was used to estimate the ancestral proportion of each individual relative to the inferred reference populations. The results were visualized using the R package pophelper.

#### 2.5.5. Screening of Candidate Genes for Twinning Trait

In this study, selection-signature detection was performed in Simmental cattle using a genome-wide sliding window approach with a window size of 50 kb (50,000 bp) and a sliding step size of 20 kb (20,000 bp). The detection process combined single-indicator analyses of population differentiation index (*F*_st_) and nucleotide diversity (θπ) with a joint analysis of both indicators to accurately screen for selection signatures. In the joint analysis of *F*_st_ and θπ, the screening criteria were as follows: windows that simultaneously fell within the top 5% of *F*_st_ values and the top 5% of θπ-ratio values were identified as genomic regions with strong selective sweep signals. They were considered selection regions for candidate loci. Furthermore, the twinning trait, as a rare biological event with extremely low incidence under natural conditions, is associated with functional genetic variants that typically exhibit a characteristic low-frequency distribution. Considering the low population frequency of such rare variants and their random distribution across the genome, the analysis strategy adopted in this study took the union of significant regions identified by the two different indicators to maximize the search scope, thereby increasing the coverage of potentially key functional loci and ensuring a more comprehensive and reliable basis for subsequent functional validation and analysis.

The population differentiation index (*F*_st_) was calculated using the sliding-window method as follows: VCFtools v0.1.17 was used with the parameters ‘--fst-window-size 50000 --fst-window-step 20000’ to calculate the *F*_st_ for each window. Regions within the top 5% of *F*_st_ values for each population pair were screened as candidate genomic regions and visualized in R. Nucleotide diversity (Pi) was calculated using VCFtools v0.1.17 software with the parameters ‘--window-pi 50000 --window-pi-step 20000’. After calculation, the ratio between each pair of groups (i.e., θπ-ratio) was calculated. The θπ-ratio values were further Log_2_-transformed to obtain Log_2_ (θπ-ratio) values, which were then ranked. Windows with Log_2_ (θπ-ratio) values in the top 5% were selected as candidate regions, and the results were visualized in R.

Based on the rare nature of the twinning trait in cattle, alleles with a population frequency below 5% were defined as low-frequency alleles in this study. We hypothesized that functional variants associated with the twinning trait may be relatively rare in general cattle populations and may be enriched in twin-bearing individuals. First, SNP loci with allele frequencies less than 0.05 in all six populations were screened. Subsequently, quality control was performed on the twin-bearing population using the following criteria: SNP loci with a minimum allele count greater than 10 and a genotype missing rate less than 0.1 (parameters set to ‘--mac 10 --max-missing 0.9’). Finally, the above loci were screened in the twin-bearing population, and candidate genes were obtained through gene annotation. It should be noted that twinning may also involve moderate-frequency variants with small-to-moderate effects, but reliable detection of such variants typically requires larger sample sizes.

#### 2.5.6. GO and KEGG Enrichment Analysis

To better understand the molecular functions of the candidate genes, GO and KEGG enrichment analyses were performed on the candidate genes from overlapping windows using the DAVID online website (https://davidbioinformatics.nih.gov/, accessed on 8 April 2026). Due to the limited number of candidate genes, a threshold of uncorrected *p* < 0.1 was set to define significant enrichment. The results were visualized using the Bioinformatics online platform (https://www.bioinformatics.com.cn/, accessed on 10 April 2026).

## 3. Results

### 3.1. Genomic Data Description

In this study, whole-genome resequencing data of 77 Simmental cattle from the Xinjiang region showed that each individual generated an average of 202 million reads, with an average mapping rate to the reference genome of 99.69% and an average sequencing depth of 10.63×. For the 79 Chinese Simmental cattle previously sequenced by our group, the average number of reads generated was 756 million, the average mapping rate was 99.75%, and the average sequencing depth was 40.46×. For the 206 cattle whole-genome resequencing data downloaded from public databases, each individual generated an average of 302 million reads, with an average mapping rate of 98.91% and an average sequencing depth of 13.31×. Detailed sequencing metrics are provided in Supplementary Table S2.

### 3.2. SNP Detection and Annotation

**Figure 1:**
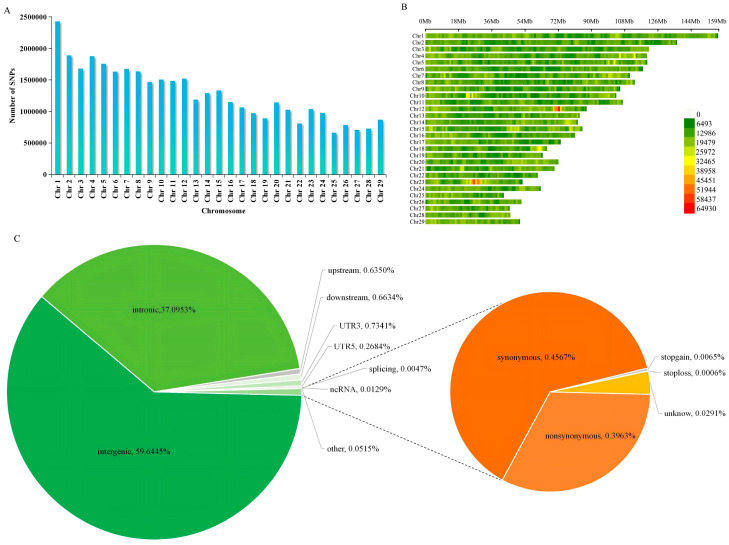
Distribution and functional annotation of SNPs in Simmental cattle. (**A**) Number distribution of SNPs on autosomes. (**B**) Distribution of SNPs on autosomes. The x-axis represents SNP density or count; the y-axis represents the positions or intervals of the 29 chromosomes; different colors indicate the number of SNPs per 1 Mb window. (**C**) Functional distribution of SNPs. The left pie chart shows the composition of SNPs with percentages labeled: intergenic, intronic, upstream, downstream, UTR3, UTR5, splicing, ncRNA, and other. The right pie chart shows the composition of exonic variants with percentages labeled: nonsynonymous, synonymous, stopgain, stoploss.

After variant detection and quality control, a total of 37,176,922 high-quality SNPs were obtained. The density distribution of SNPs on each chromosome is shown in Figure 1A,B. Among the autosomes, chromosome 1 was the longest (158.53 Mb) and contained the largest number of SNP loci (2,427,591); chromosome 25 was the shortest (42.35 Mb) and contained the smallest number of SNP loci (660,124). Functional annotation of the filtered SNPs is shown in Figure 1C. The majority of SNPs in Simmental cattle were located in intergenic regions, accounting for 59.64% of the total; followed by intronic and exonic regions, accounting for 37.10% and 0.90%, respectively.

### 3.3. Genetic Distance Matrix and Genetic Relationship Matrix Analysis

**Figure 2:**
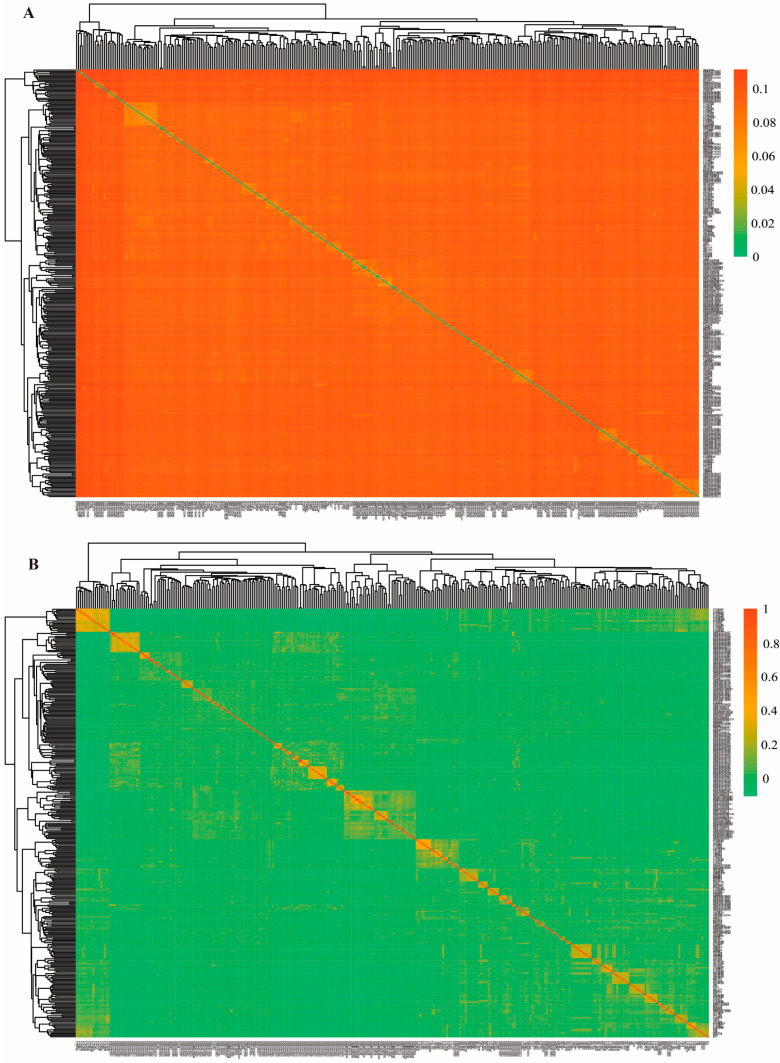
Genetic distance and genetic relationship matrices for Simmental cattle. (**A**) IBS genetic distance matrix of Simmental cattle. The horizontal and vertical axes represent individual cattle IDs, and each cell indicates the genetic distance value between two individuals. Colors closer to purple indicate greater genetic distance; conversely, colors closer to green indicate smaller genetic distance. (**B**) Genomic genetic relationship G-matrix of Simmental cattle. The horizontal and vertical axes represent individual cattle IDs, and each cell indicates the genetic relationship coefficient between two individuals. Colors closer to purple indicate closer genetic relationships; conversely, colors closer to green indicate more distant genetic relationships.

In this study, the IBS genetic distance matrix and genomic genetic relationship G-matrix of the Simmental cattle population were constructed using PLINK software, and the population genetic structure and relatedness patterns were visualized via heatmaps. The IBS genetic distance values among the 335 Simmental cattle ranged from 0.00309411 to 0.111501, with an average of 0.095. The IBS distance heatmap (Figure 2A) showed that among 55,945 unique pairwise comparisons of 335 Simmental cattle, 55,359 pairs (98.953%) had an IBS genetic distance greater than 0.08 (corresponding to the orange regions in the heatmap), indicating that the vast majority of individuals exhibited relatively distant genetic relationships and a moderate degree of kinship. Only 5 pairs (0.009%) had an IBS distance less than 0.02 (green regions), indicating that very few individual pairs shared extremely close genetic relationships. The genomic relatedness G-matrix heatmap (Figure 2B) showed a trend consistent with the IBS distance matrix: 55,904 pairs (99.927%) had G-matrix values less than 0.2 (green regions), reflecting moderate genetic relatedness among most individuals, while only 0.004% of pairs had G-matrix values greater than 0.6 (orange regions), indicating very few closely related individuals. Together, these results demonstrate that the Simmental cattle population contains only a minimal number of closely related individual pairs.

### 3.4. ROH Statistics and Inbreeding Coefficient Analysis

**Figure 3:**
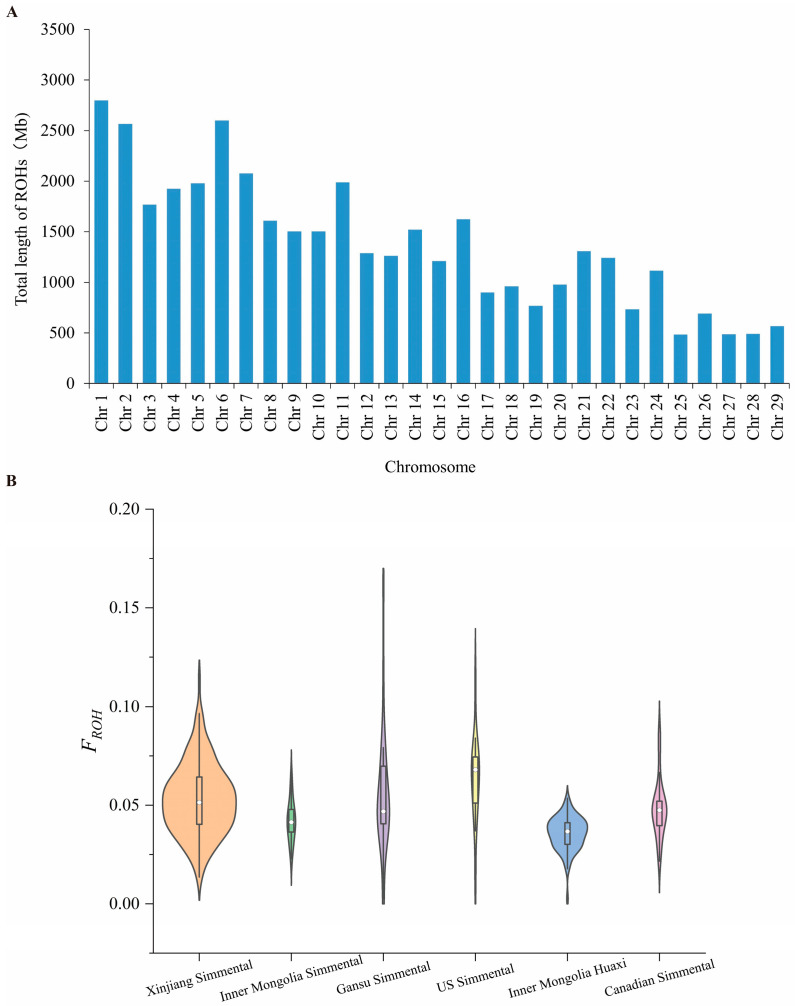
Genome-wide ROH analysis of Simmental cattle. (**A**) Length distribution of ROH on autosomes. (**B**) Inbreeding coefficient based on ROH. In the figure, Simmental cattle from Xinjiang, China, are denoted as Xinjiang Simmental; from Inner Mongolia, China, as Inner Mongolia Simmental; from Gansu, China, as Gansu Simmental; from the United States as US Simmental; Huaxi cattle from Inner Mongolia, China, as Inner Mongolia Huaxi; and Simmental cattle from Canada as Canadian Simmental. The same applies to the following figures.

A total of 48,985 ROH segments were detected in the Simmental cattle population, with a total ROH length of 39.96 Gb across the population. On average, each individual carried 147.10 ROH segments; the mean total ROH length per individual was 120.01 Mb, and the average length of a single ROH segment was 0.80 Mb. Figure 3A shows the length distribution of ROH on autosomes. In the Simmental cattle population, chromosome 1 had the longest total ROH length, while chromosome 25 had the shortest. The results of the inbreeding coefficient based on ROH (*F_ROH_*) are shown in Figure 3B. For the Xinjiang Simmental population, the individual inbreeding coefficient ranged from 0.014 to 0.114, with an average of 0.053 ± 0.018; for the Inner Mongolia Simmental population, the range was 0.023-0.065, with an average of 0.042 ± 0.011; for the Gansu Simmental population, the range was 0.003-0.161, with an average of 0.055 ± 0.031; for the US Simmental population, the range was 0.012-0.112, with an average of 0.063 ± 0.022; for the Inner Mongolia Huaxi population, the range was 0.003-0.054, with an average of 0.036 ± 0.008; and for the Canadian Simmental population, the range was 0.022-0.087, with an average of 0.048 ± 0.015.

### 3.5. Linkage Disequilibrium Analysis LD Decay Analysis

**Figure 4:**
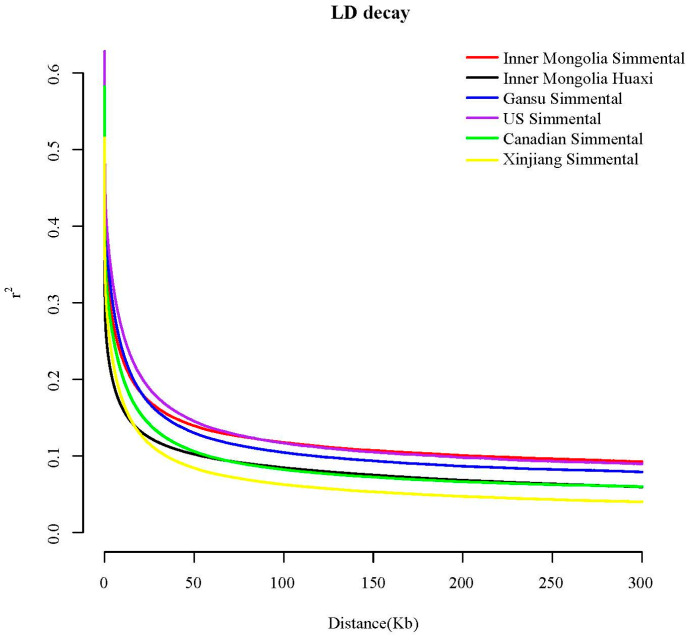
LD decay of Simmental cattle populations from different regions. The x-axis represents the physical distance over which LD is measured; the y-axis represents the LD correlation coefficient r^2^.

Figure 4 shows the linkage disequilibrium (LD) decay trends of Simmental cattle populations from different geographical origins. All populations exhibited rapid LD decay with increasing physical distance, with a sharp decline within the 0–50 kb interval, followed by a gradual flattening of the decay rate beyond 50 kb. The US Simmental population showed the slowest LD decay. In contrast, the Xinjiang Simmental population in China showed the fastest LD decay, indicating higher genetic diversity and a larger effective population size. Differences in LD decay among populations provide an important background for the subsequent detection of selection signatures.

### 3.6. Population Genetic Structure Analysis

**Figure 5:**
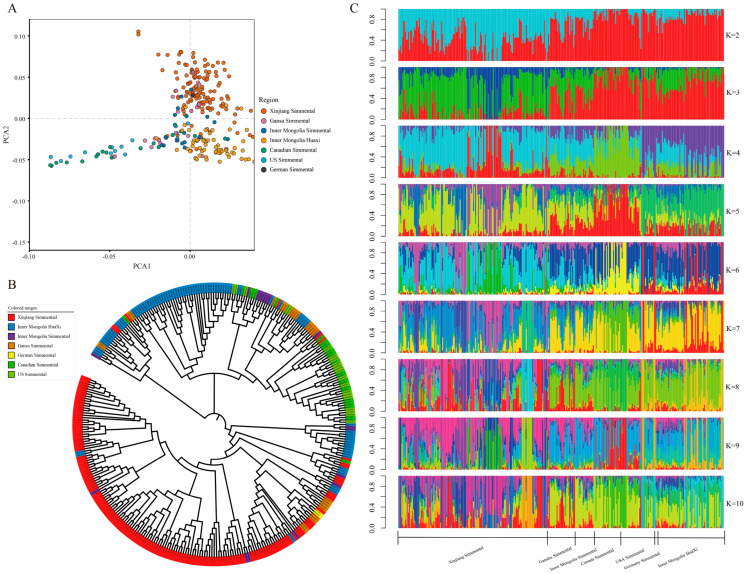
Population structure analysis of Simmental cattle. (**A**) Principal component analysis of cattle from different regions. (**B**) Phylogenetic tree of cattle from different regions. (**C**) Population structure analysis of individual cattle from different regions. Each vertical bar represents an individual; the y-axis indicates the proportion of each estimated ancestral component; different colors represent different estimated ancestral components. In the figure, Simmental cattle from Germany are denoted as German Simmental.

Principal component analysis (PCA) was performed on the Simmental cattle populations in this study. A visualization of the first two principal components (PC1 and PC2) for all individuals is shown in Figure 5A. The PCA results clearly revealed a distinct clustering pattern among Simmental cattle populations, with groups from different geographical origins exhibiting well-defined clustering characteristics, indicating clear population stratification. Simmental cattle from Xinjiang, Inner Mongolia, and Gansu in China formed a relatively concentrated cluster, whereas populations from Canada and the United States showed independent clustering tendencies. Although Huaxi cattle originated from China, they formed a separate branch in the PCA plot, suggesting certain genetic differences from the Simmental populations. To further validate the genetic relationships among populations, a phylogenetic tree was constructed using MEGA software (Figure 5B). The results were highly consistent with the PCA analysis: Chinese indigenous Simmental populations from different geographical regions (Xinjiang, Inner Mongolia, Gansu) clustered together in the phylogenetic tree; Canadian and US Simmental cattle each formed independent clusters; and Huaxi cattle constituted a separate evolutionary branch. The genetic differentiation among populations corresponded well with their geographical distributions, confirming that genetic differentiation exists among populations and may be related to factors such as geographical isolation and differences in artificial breeding directions. The population genetic structure was then analyzed using the ADMIXTURE model, with the number of ancestral populations (K) evaluated from 2 to 10. The cross-validation error results (Supplementary Table S3) showed that the CV error reached its minimum at K = 5, which was determined as the optimal number of ancestral components. At K = 5, each population exhibited clear genetic differentiation: individuals from different geographic origins displayed distinct genetic clustering patterns, while some populations simultaneously contained multiple ancestral components, indicating a certain degree of gene flow and genetic admixture among populations (Figure 5C). These results corroborate the findings from PCA and neighbor-joining tree analyses, collectively revealing the genetic structure characteristics of the Simmental cattle populations under study.

### 3.7. Selection Signature Detection Results for Twinning Trait in Chinese Simmental Cattle from Xinjiang

#### 3.7.1. Screening of Candidate Genes for Twinning Trait Selection Signature Analysis of the Twinning Trait

**Figure 6:**
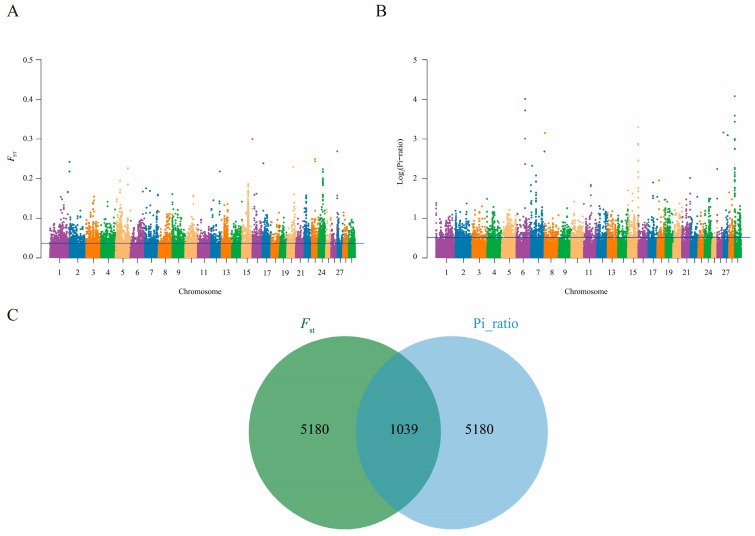
Selection signature analysis of Simmental cattle. (**A**) Genome-wide distribution of *F*_st_. (**B**) Genome-wide distribution of θπ ratio. (**C**) Venn diagram of candidate gene windows identified by the two methods. In the figure, Manhattan plot: different colors represent different chromosomes; Venn diagram: green for FST, blue for Pi-ratio.

In this study, a genome-wide sliding-window approach was used to analyze selection signatures. The population differentiation index (*F*_st_) between the twin-bearing and single-bearing groups of Xinjiang Simmental cattle was calculated and is shown in Figure 6A. The nucleotide diversity ratio (θπ ratio) between the two groups was also calculated, with the results shown in Figure 6B. Selection regions were screened using a top 5% significance threshold (*F*_st_ > 0.037 and Log_2_(θπ ratio) > 0.56), and candidate gene windows were identified through joint analysis (Figure 6C). Within the candidate intervals, SNP loci with allele frequencies ≤ 0.05 in all six Simmental populations were further screened. After stringent quality control of the twin-bearing population, a total of 286 significant SNP loci were identified, which were annotated to 89 candidate genes (Supplementary Table S4). Enrichment analysis was performed on all candidate genes. Among the candidate genes, *CYP19A1*, *HORMAD1*, *GRB14*, *CADM2*, *CXCR4*, and others have been confirmed to be associated with the twinning trait (Table 1).

#### 3.7.2. Enrichment Analysis of Candidate Genes for Twinning Trait

**Figure 7:**
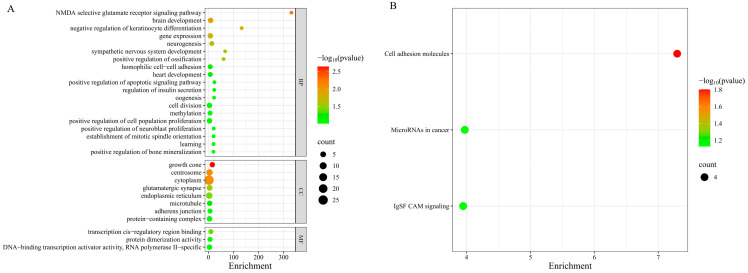
Enrichment analysis of candidate genes for twinning trait in Chinese Simmental cattle from Xinjiang. (**A**) GO enrichment analysis of candidate genes. (**B**) KEGG enrichment analysis of candidate genes.

GO and KEGG functional enrichment analyses were performed on the annotated candidate genes. The results showed that, at the biological process level, the candidate genes were mainly enriched in NMDA selective glutamate receptor signaling pathway (*p* = 0.00594), brain development (*p* = 0.0125), neurogenesis (*p* = 0.0221), positive regulation of apoptotic signaling pathway *(p* = 0.0800), positive regulation of ossification (*p* = 0.0322), and regulation of insulin secretion (*p* = 0.0828). In addition, genes were enriched in the oogenesis pathway (*p* = 0.0855), which is a core biological process for oocyte formation, maturation, and follicular development, and may influence twinning rate by affecting ovulation number. At the cellular component level, the candidate genes were enriched in subcellular structures such as growth cone (*p* = 0.00229), centrosome (*p* = 0.00813), microtubule (*p* = 0.0633), endoplasmic reticulum (*p* = 0.0367), and glutamatergic synapse (*p* = 0.0281). These structures are involved in cell division, meiosis, and signal transduction, and may participate in oocyte maturation and early embryonic development. At the molecular function level, the candidate genes were mainly associated with transcription cis-regulatory region binding (*p* = 0.0414) and DNA-binding transcription activator activity (*p* = 0.0973) (Figure 7A). KEGG pathway enrichment results (Figure 7B) showed that these genes were primarily enriched in the cell adhesion molecules pathway (*p* = 0.0158), which is involved in embryo implantation and uterine receptivity; IgSF CAM signaling (*p* = 0.0742); and microRNAs in cancer pathway (*p* = 0.0731), the latter of which may act by regulating reproduction-related gene expression.

## 4. Discussion

Population genetic diversity refers to the sum of genetic variation within and among populations of the same species. It is a core component of biodiversity and the material basis for species to adapt to environmental changes and maintain evolutionary potential. Generally, higher genetic diversity within a population indicates a stronger capacity to adapt to environmental changes and survive [20]. To further validate the characteristics of genetic relationships among populations, this study performed a combined analysis using IBS distance and G-matrix heatmaps. The IBS distance matrix heatmap showed that most individuals in the population had large genetic distances, with only a few showing close genetic relationships. The G-matrix heatmap further quantified the strength of genetic associations among individuals. The results from the two matrices were highly consistent, collectively confirming that the majority of individuals in this population exhibited moderate genetic relatedness.

ROH are important, quantifiable genetic markers at the genome level that directly reflect individual inbreeding levels, population genetic structure, and evolutionary history. Studies have shown that the inbreeding coefficient calculated from ROH (*F_ROH_*) can effectively circumvent the limitations of incomplete pedigree records and, compared with that calculated by traditional pedigree methods, more accurately reflects the actual inbreeding level of a population at the genomic level [21]. In this study, ROH analysis revealed an average inbreeding coefficient of 0.053 ± 0.0188 (range: 0.014-0.114) in Xinjiang Simmental cattle. This value is higher than the average inbreeding coefficients previously reported for Simmental cattle (0.0003) by Hu Xin et al. [22] and for South African Nguni cattle (0.033) by Maxman et al. [23], but significantly lower than the average inbreeding coefficient reported for Hereford cattle (0.229) by Sumreddee et al. [24], and is comparable to the average inbreeding coefficient reported for Holstein cattle (0.079) by Ristanic et al. [25]. These findings indicate that the inbreeding level of Xinjiang Simmental cattle is in the moderate-to-low range, with no severe accumulation of inbreeding, and the population genetic structure is relatively stable.

Linkage disequilibrium (LD) analysis can be used to identify differences in genetic diversity among populations. A faster LD decay rate indicates higher genomic genetic diversity within the population [26]. In this study, the Chinese Simmental population exhibited a faster decay rate than other populations, indicating higher genetic diversity and lower genomic selection. This result is closely related to the complex genetic background of Chinese Simmental cattle, shaped by long-term localization, involving the introduction of multiple batches of foreign Simmental bulls for crossbreeding and improvement, combined with local adaptive selection. In contrast, North American Simmental cattle have undergone long-term, high-intensity directional selection, resulting in a relatively homogeneous genetic background and lower genetic diversity [27,28], hence their slower LD decay rate. As a newly bred Chinese indigenous breed, Huaxi cattle showed an LD decay rate intermediate between those of Chinese Simmental and North American Simmental cattle. This may be related to their breeding history, which incorporated both the excellent genes of foreign Simmental cattle [29] and the genetic characteristics of local Chinese breeds, combined with a certain degree of systematic selection, reflecting their unique genetic structure and selection history.

PCA results revealed the geographic genetic differentiation in Simmental cattle worldwide. Chinese Xinjiang Simmental cattle and Chinese Huaxi cattle were independent and highly concentrated. Xinjiang Simmental cattle showed close genetic distances to domestic Simmental cattle from Gansu and Inner Mongolia. However, they exhibited significant genetic differentiation from foreign Simmental cattle from the United States, Canada, and Germany, clearly demonstrating the shaping effect of regional selection on population genetic structure. The phylogenetic tree validated the population clustering characteristics from an evolutionary perspective. Xinjiang, Gansu, and Inner Mongolia Simmental cattle formed a large, independent branch, clearly separated from foreign Simmental cattle (United States, Canada), while Huaxi cattle formed an independent evolutionary branch. Within this framework, Xinjiang Simmental cattle clustered with other domestic Simmental populations, further supporting the close genetic relationships among them, consistent with the breeding history of introduction and improvement of domestic Simmental cattle. Meanwhile, foreign Simmental cattle from the United States and Canada clustered into independent branches, reflecting the distinct genetic characteristics formed by long-term directional selection. Although ADMIXTURE has known limitations when applied to unevenly sampled or closely related populations, our cross-validation results confirmed K = 5 as the optimal model, and the inferred genetic structure was highly consistent with PCA and neighbor-joining tree analyses [30]. The population structure analysis results confirmed K = 5 as the optimal clustering model, revealing a clear genetic differentiation pattern among different Simmental cattle populations, which is highly consistent with their geographic origins and breeding history. The unique genetic components of US Simmental and German Simmental reflect their distinct genetic backgrounds shaped under different selection systems. In contrast, the shared ancestral components between Inner Mongolia Simmental and Canadian Simmental suggest a common origin or extensive gene flow between them. Xinjiang Simmental and Inner Mongolia Huaxi exhibited highly homogeneous genetic compositions at K = 5, indicating that their genetic backgrounds are relatively simple and have experienced less introgression from other populations.

The twinning trait is an important indicator of reproductive performance in cattle, directly affecting cow reproductive efficiency and farm economic benefits. Cattle are typical uniparous livestock, with an extremely low natural twinning rate. Although twin pregnancy can effectively increase the annual number of calves per cow and reduce unit production costs, it also significantly increases the risk of reproductive disorders such as abortion, dystocia, stillbirth, retained placenta, and postpartum metabolic disturbances [31]. Because the twinning trait in cattle is a low-heritability trait [32,33], and its phenotype is easily influenced by non-genetic factors such as feeding management and environmental conditions, genetic progress through traditional breeding methods has been slow [34]. In recent years, with the rapid development of genome-wide association studies (GWAS), selection signature detection, and quantitative trait locus (QTL) mapping technologies, a series of key candidate genes regulating multiple-birth traits in livestock have been successfully identified. Their functions involve multiple biological processes, including reproductive hormone synthesis and secretion, follicular development and maturation, regulation of ovulation, embryo implantation, and maintenance of pregnancy [35]. Therefore, how to effectively increase the twinning rate in cattle while avoiding the reproductive risks associated with twinning remains a research hotspot and key challenge in beef and dairy cattle breeding.

In this study, Chinese Simmental cattle were used as the research subjects to perform genome-wide selection-signature analysis for the twinning trait and to identify functional enrichment of candidate genes, providing new clues for an in-depth understanding of the genetic mechanisms underlying the twinning trait in cattle. By conducting selection signature analysis on 57 Simmental cattle with twin pregnancy records, a total of 89 candidate genes were ultimately screened, including *CYP19A1*, *HORMAD1*, *GRB14*, *CADM2*, *CXCR4*, and others that have been confirmed to be associated with the twinning trait. *HORMAD1* belongs to the HORMA domain (Hop1p, Rev7p, and MAD2) family [36] and is involved in the formation of the meiotic chromosome axis, which is essential for gametogenesis in mammals [37]. It has been confirmed that *HORMAD1* and its family members are highly expressed in testicular tissue, are lowly expressed in other tissues, and are overexpressed in cancerous tissues [38]. Recent studies have shown that the *HORMAD1* protein is a key regulator during mammalian gametogenesis, acting as a supervisor during meiosis and coordinating other reproduction-related proteins via distinct phosphorylation patterns to complete the meiotic process [39]. Shin et al. [40] used gene expression profiling and found that *HORMAD1*-knockout mice, both male and female, exhibited infertility, demonstrating the necessity of *HORMAD1* for mammalian gametogenesis. Furthermore, Alhathal et al. [41] reported a significant association (moderate evidence level) between missense variants in the *HORMAD1* gene and non-obstructive azoospermia in a large-scale male infertility cohort study. This study confirmed that mutations in the *HORMAD1* gene directly impair spermatogenesis, clinically presenting as azoospermia or severe oligospermia, further validating the critical role of this gene in male reproduction. Additionally, enrichment analysis showed that this gene was significantly enriched in the oogenesis signaling pathway, further suggesting its important function in reproduction. *GRB14*, a member of the *GRB7* family, encodes an adaptor protein containing an SH2 domain that negatively regulates receptor tyrosine kinase signaling pathways and participates in metabolic regulation and cell proliferation. In this study, this gene was significantly enriched in the cytoplasmic structure, suggesting that it may influence cell proliferation, differentiation, or metabolic processes related to reproductive traits by regulating relevant signaling pathways within the cytoplasm. Bohrer et al. [42] showed that during bovine follicular development, *GRB14* is expressed in both granulosa and theca cells of follicles at all stages. During the follicular dominance selection phase, its mRNA expression level was significantly higher in subordinate follicle granulosa cells than in dominant follicle granulosa cells (*p* < 0.05), suggesting that *GRB14* may negatively regulate follicular dominance selection. Zhao et al. [43] first reported, through differential expression analysis of follicles from Meishan and Duroc pigs, a potential association between *GRB14* and reproductive traits in pigs. The study found that the *GRB14* gene was highly expressed in reproductive organs, including the ovary, oviduct, corpus luteum, and pituitary. In contrast, its expression was low in non-reproductive organs, suggesting that this gene may have a specific function in reproductive tissues. Moreover, its mRNA abundance was significantly negatively correlated with both P450Arom (aromatase) mRNA in granulosa cells and estradiol concentration in follicular fluid, suggesting that *GRB14* influences follicular differentiation by inhibiting estrogen synthesis. In chickens, circGRB14 has been shown to inhibit granulosa cell proliferation and promote apoptosis by sponging specific microRNAs, thereby indirectly affecting oocyte developmental competence by regulating the follicular microenvironment [44]. Consistent with its enrichment in the cytoplasm (GO:0005737), *GRB14* may mediate intracellular signaling cascades that influence granulosa cell proliferation and follicular selection, thereby potentially affecting ovulation rate and twinning. In addition, the *CYP19A1* gene, which is closely related to reproductive traits, was identified approximately 70 kb upstream of a significant SNP locus in this study. The *CYP19A1* gene, also known as aromatase, is a key enzyme in estrogen biosynthesis. It is a member of the cytochrome P450 superfamily and is encoded by the *CYP19A1* gene. This gene is expressed to varying degrees in the ovary, placenta, pituitary, and other tissues. It is a biosynthetic enzyme that catalyzes the conversion of androgens into different forms of estrogen in vertebrates, serving as a critical rate-limiting enzyme in estrogen synthesis [45]. The aromatase protein P450 is a type of cytochrome enzyme composed of hemoglobin and luteal protein, acting as the rate-limiting and key enzyme that converts androgens into estrogens. It aromatizes the A ring of androgens, removes the carbon atom at position 19, and converts carbonyl groups to hydroxyl groups, yielding estrone and estradiol from androstenedione and testosterone, respectively. Localization analysis of *CYP19A1* expression during different stages of follicular development and oocyte maturation in yaks suggested that *CYP19A1* is involved in and promotes folliculogenesis and oocyte formation, revealing that *CYP19A1* plays a positive regulatory role in both follicular development and oocyte maturation during the reproductive process of female yaks [46]. Using QRT-PCR, mRNA expression of the *CYP19A1* gene was detected in various buffalo tissues, and the results showed substantial differences in *CYP19A1* expression among tissues [47]. Zhang et al. [48] found that this gene is widely expressed in follicles, corpora lutea, and granulosa cells in goats. Subcellular localization results showed that its encoded protein is mainly localized in the cytoplasm and nucleus, suggesting that it is involved in intracellular hormone synthesis and may also indirectly participate in transcriptional regulation. Functional validation experiments further confirmed that overexpression of *CYP19A1* significantly upregulated the mRNA expression levels of FSHR (follicle-stimulating hormone receptor) and INHBA (inhibin subunit alpha) in granulosa cells, while promoting granulosa cell proliferation and the secretion of estrogen and progesterone. Conversely, silencing *CYP19A1* expression led to opposite phenotypic changes. The study proposed that CYP19A1 may influence litter size traits in female livestock by regulating granulosa cell proliferation, reproductive hormone secretion, and the expression networks of multiple-birth-related candidate genes. Echternkamp et al. [49] further demonstrated in twin-bearing cattle that *CYP19A1* mRNA expression levels in granulosa cells were significantly higher than those in control cows, and this upregulation acted synergistically with increased FSHR expression to constitute the molecular basis for increased ovulation rate. Twin-bearing cows had significantly elevated IGF-1 concentrations in blood and follicular fluid, and IGF-1 stimulated follicular development through the IGF-1R signaling pathway. Meanwhile, downregulation of IGF-2R expression increased the availability of free IGF-2, and the two synergistically promoted follicular development. *CYP19A1* enrichment in the endoplasmic reticulum (GO:0005783) reflects its function in estrogen production, a key regulator of folliculogenesis and ovulation, thereby potentially affecting twinning rate. *CXCR4* is the receptor for the chemokine *CXCL12*. *CXCL12* produced by the embryo binds to *CXCR4* in uterine tissues, forming the CXCL12-CXCR4 signaling axis. This signaling axis is closely related to tissue remodeling, including angiogenesis, which is essential for the structural and functional transformations required for the uterus to accept the embryo and for establishing uterine receptivity. This signaling axis plays an important role in early pregnancy, participating in the dialogue between the embryo and the uterus and influencing embryo survival and the establishment of pregnancy. Polymorphisms in the *CXCR4* gene have been reported to be associated with uterine receptivity and fertility in cattle, providing a direction for screening genetic markers to improve reproductive efficiency [50]. *CXCR4* is enriched in brain development, neurogenesis, and protein-containing complex pathways (GO:0007420, GO:0022008, GO:0032991), reflecting its roles in cell migration and protein interactions, processes also critical for embryo implantation, thereby potentially influencing twinning. *CADM2* (Cell Adhesion Molecule 2) has been identified as a candidate gene for reproductive traits in buffalo. A SNP (AX-85077363) downstream of the *CADM2* gene was found to be associated with days to second calving [51]. Wu et al., using a bovine 50K SNP chip in a GWAS study on buffalo, identified a SNP located upstream of the *CADM2* gene associated with the interval between first and second calving in buffalo [52]. *CADM2* is enriched in homophilic cell–cell adhesion (GO:0007156) and IgSF CAM signaling pathways (bta04517), reflecting its role in intercellular recognition and adhesion, processes that are critical for follicular development and uterine receptivity, thereby potentially affecting twinning.

In this study, control individuals were defined as cows with at least four parities and no recorded twin births. However, because the twinning trait is strongly influenced by environmental factors (e.g., nutrition, stress, season) and stochastic events, some individuals carrying twinning-predisposing genes may fail to express the twin phenotype due to environmental conditions. Similarly, a study on the polytocous trait in Tibetan sheep, which performed GWAS and multi-omics analyses on multi-lamb (twin/triplet) versus single-lamb individuals across three consecutive lambing seasons, also found that the polytocous trait is regulated by both genetic and environmental factors and identified key genes such as *BMPR1B* and *PAPPA* [53]. Given the low heritability and complexity of the twinning trait, the control group in this study may contain a small number of individuals genetically predisposed to twinning but phenotypically negative, which increases the risk of false-negative or false-positive findings-a common limitation in twinning research. To mitigate this impact, we took the following measures: (1) requiring control cows to have at least four parities to improve phenotypic reliability; and (2) matching parity distribution between the twin and control groups to control for parity effects. Furthermore, for the low-frequency variant screening associated with the twinning trait, the allele count threshold used in this study corresponded to a minor allele frequency slightly above the conventional rare-variant definition (5%). This was an exploratory compromise between false-positive control and retention of true signals under the current sample size: applying a more stringent frequency threshold dramatically increased the number of candidate genes, most of which lacked known associations with reproductive functions, and the enrichment analysis became diffuse without core pathways. Therefore, our screening strategy was intentionally conservative to prioritize reducing false positives. In summary, the candidate genes identified in this study need to be further validated through larger sample sizes, prospective designs, more systematic parameter optimization strategies (e.g., stricter rare-variant thresholds), and functional validation experiments.

## 5. Conclusions

Based on whole-genome resequencing data and global Simmental cattle genomic information from public databases, this study systematically analyzed the genetic structure and selection signatures of populations from different geographical origins. Population genetic analysis revealed that Simmental cattle from various regions worldwide generally exhibit moderate genetic relatedness, with Chinese Simmental cattle and Huaxi cattle showing clear genetic differentiation from other populations. The Xinjiang Simmental population exhibited the fastest rate of linkage disequilibrium (LD) decay, with a mean *F_ROH_* of 0.053, suggesting abundant genetic diversity. Ancestral component analysis showed that K = 5 was the optimal model, clearly elucidating the genetic differentiation and ancestry composition differences among Simmental and Huaxi cattle populations. Furthermore, selection signature analysis of the twinning trait, by comparing genomic differences between twin-bearing and single-bearing individuals, identified 89 candidate genes, including *CYP19A1*, *HORMAD1*, *GRB14*, *CADM2*, *CXCR4*, and others reported to be closely associated with the twinning trait. This study clarifies the population genetic structure of Chinese Simmental cattle in the Xinjiang region, providing a theoretical basis and candidate molecular markers for in-depth analysis of the genetic mechanisms underlying the twinning trait and for molecular breeding of high fecundity.

## Figures and Tables

**Table 1:** Candidate genes associated with twinning trait in Simmental cattle.

Candidate Gene	SNP	Allele Frequency	Allele Frequency in Simmental Control Population
*HORMAD1*	3:20018475, 3:20020067, 3:20020145, 3:20022666, 3:20022817, 3:20023189, 3:20023194, 3:20024918, 3:20024954, 3:20025156, 3:20025487, 3:20025739, 3:20026543, 3:20026940, 3:20027337, 3:20027383, 3:20027691, 3:20029358, 3:20029932, 3:20030293, 3:20030812, 3:20031386, 3:20018475, 3:20020067, 3:20020145, 3:20022666, 3:20022817, 3:20023189, 3:20023194, 3:20024918, 3:20024954, 3:20025156, 3:20025487, 3:20025739, 3:20026543, 3:20026940, 3:20027337, 3:20027383, 3:20027691, 3:20029358, 3:20029932, 3:20030293, 3:20030812, 3:20031386, 3:20032664, 3:20034510	0.0877193~0.0982143	0~0.0470588
*GRB14*	2:32594609	0.0877193	0~0.0192308
*CYP19A1*	10:59233183	0.122807	0~0.037037
*CADM2*	2:60733735, 2:60746335	0.0877193	0~0.0470588
*CXCR4*	2:33419929	0.0877193	0~0.0277778

## Data Availability

The data and materials used in this research are available from the corresponding author upon request.

## Supplementary Materials

The following supporting information can be downloaded at https://www.mdpi.com/article/10.3390/ani16101567/s1. Table S1. Sample composition and population assignment of Simmental and Huaxi cattle used in this study. Table S2. Summary statistics of BAM files for Simmental cattle. Table S3. Cross-validation errors for each K value in Simmental cattle populations from different regions. Table S4. 286 SNP loci significantly associated with twinning trait in Chinese Simmental cattle from Xinjiang region.
