# Supplementary material for: Population Structure Analysis and Candidate Gene Screening for Twinning Trait in Simmental Cattle
Source: Animals (Basel). 2026 May 21;16(10):1567. doi: 10.3390/ani16101567 (PMC13203148; doi:10.3390/ani16101567)
Supplement: Supplementary file 1 [file animals-16-01567-s001.zip › animals-4299702-supplementary.pdf]

Table S1. Sample composition and population assignment of Simmental and Huaxi cattle used in this study

Population	Geographic origin	Data source	Number of individuals	Overlap with other sources
Xinjiang Simmental	Xinjiang, China	Newly sequenced in this study	77	None
Xinjiang Simmental	Xinjiang, China	Previously sequenced (our laboratory)	79	None
Gansu Simmental	Gansu, China	NCBI	26	None
Inner Mongolia Simmental	Inner Mongolia, China	NCBI	18	None
US Simmental	United States	NCBI	17	None
Canadian Simmental	Canada	NCBI	31	None
German Simmental	Germany	NCBI	2	None
Huaxi	Inner Mongolia, China	NCBI	85	None
Unknown origin Simmental	Unknown	NCBI	27	None
	Total		362	

Note: The 77 samples from "newly sequenced in this study" and the 79 samples from "previously sequenced (our laboratory)" are all Simmental cattle from Xinjiang, representing distinct individuals with no overlap. The 206 NCBI-downloaded samples have no overlap with the above sequencing data. The two German Simmental samples were used for whole-genome data description, SNP detection, and population genetic structure analysis, but were not included in cross-population allele frequency comparisons. The 27 Simmental cattle of unknown origin were used only for whole-genome data description, SNP detection, and annotation, and were excluded from subsequent population genetic structure analyses (PCA, Admixture, phylogenetic tree), allele frequency comparisons, and selection signature analyses.

Table S2. Summary statistics of BAM files for Simmental cattle

Sample ID	Total reads	Mapped reads	Mapping rate	Total sequenced bases (bp)	GC content	Mean depth	Instrument
1054	202,513,280	202,077,486	99.78%	29,546,318,621	44.00%	10.713X	DNBSEQ-T7
1328	211,683,100	211,238,234	99.79%	30,850,463,024	43.63%	11.1859X	DNBSEQ-T7
1357	207,548,812	207,173,127	99.82%	30,368,310,144	43.66%	11.0114X	DNBSEQ-T7
1373	191,598,812	191,184,814	99.78%	27,771,694,467	43.74%	10.0696X	DNBSEQ-T7
1522	201,606,164	201,263,187	99.83%	29,561,269,064	43.71%	10.7186X	DNBSEQ-T7
180294	198,075,748	197,494,552	99.71%	28,867,253,974	43.23%	10.4669X	DNBSEQ-T7
190042	245,451,159	245,119,207	99.86%	35,462,336,934	43.31%	12.858X	DNBSEQ-T7
190044	199,271,799	198,940,620	99.83%	28,988,113,250	43.29%	10.5108X	DNBSEQ-T7
191212	193,691,346	193,331,361	99.81%	28,225,537,653	43.60%	10.2341X	DNBSEQ-T7
192002	196,425,238	196,019,572	99.79%	28,611,208,361	43.37%	10.374X	DNBSEQ-T7
192018	203,116,196	201,828,372	99.37%	29,369,108,985	43.53%	10.6489X	DNBSEQ-T7
200083	203,659,561	200,366,062	98.38%	29,040,382,990	43.33%	10.5294X	DNBSEQ-T7
200802	204,232,508	203,323,449	99.55%	28,526,564,604	42.42%	10.3432X	DNBSEQ-T7
201048	191,566,183	191,078,000	99.75%	27,817,996,101	43.05%	10.0863X	DNBSEQ-T7
201102	192,393,892	192,003,779	99.80%	28,105,354,488	43.88%	10.1906X	DNBSEQ-T7
201256	198,167,524	197,747,026	99.79%	28,957,042,714	43.67%	10.4995X	DNBSEQ-T7
201325	228,409,494	228,047,208	99.84%	33,286,633,338	43.83%	12.0691X	DNBSEQ-T7
201353	205,071,873	204,261,838	99.60%	29,374,623,977	43.79%	10.6512X	DNBSEQ-T7
201391	198,198,859	197,842,536	99.82%	28,943,133,834	43.84%	10.4946X	DNBSEQ-T7
201414	198,542,542	197,763,166	99.61%	28,925,119,446	43.75%	10.4879X	DNBSEQ-T7
201666	227,329,040	226,938,263	99.83%	33,135,234,901	43.77%	12.0143X	DNBSEQ-T7
201704	194,193,721	193,819,092	99.81%	28,317,494,949	43.79%	10.2676X	DNBSEQ-T7

Sample ID	Total reads	Mapped reads	Mapping rate	Total sequenced bases (bp)	GC content	Mean depth	Instrument
201810	200,563,930	200,198,556	99.82%	28,696,091,896	43.93%	10.4047X	DNBSEQ-T7
202062	195,844,824	195,437,014	99.79%	28,517,158,449	43.68%	10.3397X	DNBSEQ-T7
202092	189,613,681	189,194,008	99.78%	27,633,901,877	43.88%	10.0197X	DNBSEQ-T7
202314	188,687,079	188,226,956	99.76%	27,558,865,250	44.00%	9.9924X	DNBSEQ-T7
202420	198,776,655	198,442,736	99.83%	29,025,188,723	43.80%	10.5242X	DNBSEQ-T7
202510	212,908,123	212,571,923	99.84%	31,172,447,661	43.58%	11.303X	DNBSEQ-T7
210018	198,823,144	198,166,078	99.67%	28,849,999,056	43.38%	10.4608X	DNBSEQ-T7
210022	203,901,164	203,591,012	99.85%	29,700,481,354	43.29%	10.7691X	DNBSEQ-T7
211062	195,522,521	195,037,738	99.75%	28,287,083,891	43.04%	10.2565X	DNBSEQ-T7
211084	205,465,710	204,934,308	99.74%	28,905,517,510	43.42%	10.4805X	DNBSEQ-T7
211162	214,712,951	214,296,318	99.81%	31,109,599,204	42.58%	11.2796X	DNBSEQ-T7
211174	192,155,731	191,813,769	99.82%	27,963,214,160	43.55%	10.139X	DNBSEQ-T7
211186	207,901,681	207,481,052	99.80%	29,532,447,234	43.72%	10.7078X	DNBSEQ-T7
212010	192,029,541	191,369,421	99.66%	28,027,847,136	43.83%	10.1624X	DNBSEQ-T7
212038	191,775,837	187,877,502	97.97%	27,039,894,304	43.59%	9.8046X	DNBSEQ-T7
212046	213,526,629	213,185,836	99.84%	31,205,289,976	43.78%	11.3145X	DNBSEQ-T7
212245	199,532,226	199,222,468	99.84%	29,221,958,818	43.73%	10.5955X	DNBSEQ-T7
212347	195,953,766	195,549,866	99.79%	28,457,323,250	43.82%	10.3183X	DNBSEQ-T7
212445	184,984,209	184,379,209	99.67%	26,996,581,611	43.74%	9.7885X	DNBSEQ-T7
212564	208,855,072	208,560,626	99.86%	30,377,959,596	43.90%	11.0147X	DNBSEQ-T7
212570	193,957,361	193,695,737	99.87%	28,393,889,635	43.80%	10.2953X	DNBSEQ-T7
212636	198,729,293	198,293,641	99.78%	28,715,605,377	43.86%	10.4121X	DNBSEQ-T7
212669	198,478,106	198,059,567	99.79%	28,761,162,908	43.76%	10.4287X	DNBSEQ-T7

Sample ID	Total reads	Mapped reads	Mapping rate	Total sequenced bases (bp)	GC content	Mean depth	Instrument
212835	219,936,265	219,553,735	99.83%	32,209,613,751	43.68%	11.6786X	DNBSEQ-T7
212849	222,345,753	221,649,338	99.69%	32,148,050,422	43.88%	11.6563X	DNBSEQ-T7
212992	231,618,342	226,495,575	97.79%	32,126,953,933	44.11%	11.6486X	DNBSEQ-T7
213560	197,319,242	196,789,361	99.73%	28,768,142,078	43.75%	10.4309X	DNBSEQ-T7
22	196,933,472	196,523,850	99.79%	28,763,407,063	43.71%	10.4293X	DNBSEQ-T7
221058	198,926,883	198,507,534	99.79%	28,979,236,722	43.65%	10.5075X	DNBSEQ-T7
221070	190,349,632	188,142,919	98.84%	26,611,392,558	43.16%	9.6488X	DNBSEQ-T7
224114	192,356,742	191,961,192	99.79%	28,081,984,110	43.87%	10.1821X	DNBSEQ-T7
224890	195,253,444	194,900,626	99.82%	28,329,432,935	43.77%	10.2721X	DNBSEQ-T7
390	185,724,279	185,346,880	99.80%	26,900,629,057	43.65%	9.7539X	DNBSEQ-T7
455	214,777,058	214,159,711	99.71%	30,919,437,949	43.88%	11.2109X	DNBSEQ-T7
461	197,560,785	197,175,812	99.81%	28,669,317,465	43.87%	10.3952X	DNBSEQ-T7
523	186,503,738	186,149,023	99.81%	27,277,962,697	43.68%	9.8906X	DNBSEQ-T7
528	195,043,295	194,624,044	99.79%	28,287,527,382	43.82%	10.2567X	DNBSEQ-T7
534	192,846,431	192,487,849	99.81%	28,168,151,451	43.82%	10.2134X	DNBSEQ-T7
60034	245,812,397	245,394,324	99.83%	35,837,905,413	43.94%	12.9946X	DNBSEQ-T7
61248	190,012,332	189,643,214	99.81%	27,802,981,188	44.03%	10.081X	DNBSEQ-T7
62636	184,828,606	184,455,536	99.80%	26,984,622,242	43.77%	9.7842X	DNBSEQ-T7
62807	201,489,217	201,057,710	99.79%	29,443,663,330	43.77%	10.6758X	DNBSEQ-T7
70284	203,703,567	203,098,808	99.70%	28,845,772,688	44.13%	10.459X	DNBSEQ-T7
70340	236,681,843	235,745,615	99.60%	34,211,625,092	43.78%	12.4052X	DNBSEQ-T7
70561	209,541,277	208,936,208	99.71%	30,243,583,523	44.04%	10.9657X	DNBSEQ-T7
70627	191,856,491	191,563,332	99.85%	27,553,632,141	43.95%	9.9906X	DNBSEQ-T7

Sample ID	Total reads	Mapped reads	Mapping rate	Total sequenced bases (bp)	GC content	Mean depth	Instrument
780	191,261,466	190,732,793	99.72%	27,631,120,925	44.04%	10.0186X	DNBSEQ-T7
800112	200,685,237	199,965,802	99.64%	29,279,971,078	43.58%	10.6166X	DNBSEQ-T7
800681	190,850,121	190,287,800	99.71%	27,641,844,069	44.01%	10.0225X	DNBSEQ-T7
800853	210,433,737	209,955,088	99.77%	30,473,725,336	43.96%	11.0492X	DNBSEQ-T7
800864	200,719,911	200,341,703	99.81%	29,371,086,351	43.86%	10.6498X	DNBSEQ-T7
800963	214,385,838	214,014,090	99.83%	31,066,203,999	43.95%	11.2641X	DNBSEQ-T7
801011	203,601,867	203,072,300	99.74%	29,676,386,758	43.70%	10.7605X	DNBSEQ-T7
871	196,972,531	196,520,273	99.77%	28,754,874,922	43.72%	10.4261X	DNBSEQ-T7
90021	206,269,267	205,784,811	99.77%	30,079,948,569	43.79%	10.9064X	DNBSEQ-T7
00412A	880,412,944	878,758,083	99.81%	130,536,286,800	42.74%	47.3285X	DNBSEQ-T7
00533A	1,061,440,903	1,057,699,530	99.65%	156,918,940,127	43.05%	56.8936X	DNBSEQ-T7
00621A	695,291,091	693,971,566	99.81%	102,824,708,873	42.88%	37.2809X	DNBSEQ-T7
01086B	1,166,427,965	1,164,360,305	99.82%	168,444,408,288	43.56%	61.0715X	DNBSEQ-T7
01168A	711,590,397	710,259,977	99.81%	105,523,771,054	42.57%	38.2599X	DNBSEQ-T7
01417A	864,532,884	861,232,795	99.62%	127,950,434,646	42.92%	46.3906X	DNBSEQ-T7
01464A	615,234,633	613,970,233	99.79%	91,223,909,018	42.39%	33.0748X	DNBSEQ-T7
01504A	885,235,717	883,528,200	99.81%	130,978,360,849	42.97%	47.4887X	DNBSEQ-T7
140352A	770,563,141	768,598,342	99.75%	114,147,773,295	42.69%	41.3866X	DNBSEQ-T7
150142A	700,740,447	699,123,825	99.77%	103,519,594,224	42.79%	37.533X	DNBSEQ-T7
201053B	709,367,789	706,854,291	99.65%	104,952,106,921	42.43%	38.0529X	DNBSEQ-T7
201465A	793,035,025	791,659,964	99.83%	117,627,245,074	42.74%	42.6479X	DNBSEQ-T7
201491A	682,260,398	680,781,617	99.78%	101,066,417,910	42.70%	36.6435X	DNBSEQ-T7
201580A	797,728,720	795,291,443	99.69%	118,068,958,640	42.75%	42.8082X	DNBSEQ-T7

Sample ID	Total reads	Mapped reads	Mapping rate	Total sequenced bases (bp)	GC content	Mean depth	Instrument
201632A	611,213,979	608,794,120	99.60%	90,388,591,456	42.98%	32.7722X	DNBSEQ-T7
201634A	570,425,697	569,075,066	99.76%	84,569,349,922	43.18%	30.662X	DNBSEQ-T7
201687A	632,045,868	630,951,170	99.83%	93,058,535,262	43.89%	33.7399X	DNBSEQ-T7
201733A	687,095,752	685,743,741	99.80%	101,916,282,321	42.85%	36.9516X	DNBSEQ-T7
201892A	625,108,698	623,874,974	99.80%	92,340,413,377	43.02%	33.4795X	DNBSEQ-T7
201915A	627,782,169	626,409,456	99.78%	92,975,326,044	43.11%	33.7099X	DNBSEQ-T7
201962A	598,272,423	597,035,923	99.79%	88,662,861,172	43.24%	32.1463X	DNBSEQ-T7
202237A	643,176,732	641,985,517	99.81%	95,327,982,936	42.73%	34.5635X	DNBSEQ-T7
202245A	571,747,411	569,951,456	99.69%	84,584,272,845	43.01%	30.6676X	DNBSEQ-T7
202253A	714,460,386	712,648,513	99.75%	105,499,847,974	42.84%	38.2507X	DNBSEQ-T7
202293A	641,467,764	639,470,949	99.69%	94,886,707,995	42.73%	34.4032X	DNBSEQ-T7
202326A	697,155,339	695,585,745	99.77%	103,231,885,567	42.91%	37.4286X	DNBSEQ-T7
202342A	620,416,533	618,558,145	99.70%	91,856,231,769	42.85%	33.304X	DNBSEQ-T7
202363A	616,719,904	614,040,224	99.57%	91,195,335,751	42.83%	33.0644X	DNBSEQ-T7
202468A	620,363,444	618,874,642	99.76%	92,001,245,316	42.69%	33.3568X	DNBSEQ-T7
202530A	997,757,748	996,214,817	99.85%	136,620,278,030	44.23%	49.533X	DNBSEQ-T7
202692A	744,856,990	743,705,717	99.85%	110,558,645,619	42.63%	40.0852X	DNBSEQ-T7
212048A	777,152,888	774,332,769	99.64%	114,947,992,757	43.19%	41.6763X	DNBSEQ-T7
212128A	922,771,620	921,109,366	99.82%	136,716,699,426	42.82%	49.5693X	DNBSEQ-T7
212218A	613,806,093	612,001,600	99.71%	90,937,170,875	43.09%	32.9706X	DNBSEQ-T7
212256B	721,833,917	720,442,771	99.81%	107,085,867,681	42.91%	38.8259X	DNBSEQ-T7
212364A	798,086,211	796,710,572	99.83%	118,157,346,831	42.74%	42.8405X	DNBSEQ-T7
212441A	782,313,669	781,067,256	99.84%	116,031,658,804	42.64%	42.0693X	DNBSEQ-T7

Sample ID	Total reads	Mapped reads	Mapping rate	Total sequenced bases (bp)	GC content	Mean depth	Instrument
212499A	890,941,449	889,358,800	99.82%	129,554,709,724	43.87%	46.9718X	DNBSEQ-T7
212513A	660,259,807	658,613,486	99.75%	97,893,756,932	42.75%	35.4932X	DNBSEQ-T7
212565A	694,260,219	691,928,498	99.66%	102,742,800,569	43.14%	37.2511X	DNBSEQ-T7
212629A	607,544,676	604,931,045	99.57%	89,825,791,168	42.90%	32.5679X	DNBSEQ-T7
212748A	756,771,737	753,457,468	99.56%	111,862,514,104	42.61%	40.5579X	DNBSEQ-T7
212768A	763,205,557	760,886,738	99.70%	113,035,661,222	42.80%	40.9831X	DNBSEQ-T7
212784A	760,574,918	759,107,484	99.81%	112,831,539,095	42.45%	40.9092X	DNBSEQ-T7
212789A	820,497,969	818,849,838	99.80%	121,739,041,332	42.77%	44.1387X	DNBSEQ-T7
212790A	636,202,538	634,391,266	99.72%	94,265,653,291	42.76%	34.1778X	DNBSEQ-T7
212797A	771,751,399	770,404,221	99.83%	114,423,412,226	43.04%	41.4861X	DNBSEQ-T7
212798A	754,697,398	753,175,284	99.80%	111,819,751,743	42.72%	40.5425X	DNBSEQ-T7
212812A	700,735,424	699,458,426	99.82%	103,710,868,869	42.94%	37.6021X	DNBSEQ-T7
212821A	861,447,076	859,657,980	99.79%	127,723,229,145	42.84%	46.3084X	DNBSEQ-T7
212827A	784,123,180	782,671,840	99.81%	116,339,898,768	42.90%	42.1812X	DNBSEQ-T7
212829A	894,733,001	893,404,026	99.85%	132,528,171,247	43.06%	48.0502X	DNBSEQ-T7
212878A	815,414,189	813,474,038	99.76%	120,790,989,051	42.76%	43.7951X	DNBSEQ-T7
212979A	1,031,909,183	1,029,018,829	99.72%	152,967,634,317	42.94%	55.4611X	DNBSEQ-T7
213028A	672,240,686	670,759,510	99.78%	99,592,473,484	42.89%	36.109X	DNBSEQ-T7
213038A	782,338,014	780,731,158	99.79%	115,847,079,107	42.83%	42.0024X	DNBSEQ-T7
213040A	1,277,113,590	1,274,888,208	99.83%	184,278,971,427	43.69%	66.8127X	DNBSEQ-T7
50750A	714,483,282	713,315,942	99.84%	106,062,269,433	42.86%	38.4548X	DNBSEQ-T7
60294A	1,052,919,582	1,050,664,267	99.79%	155,335,777,262	43.49%	56.3199X	DNBSEQ-T7
60343A	754,651,706	752,456,467	99.71%	111,671,470,025	42.75%	40.4884X	DNBSEQ-T7

Sample ID	Total reads	Mapped reads	Mapping rate	Total sequenced bases (bp)	GC content	Mean depth	Instrument
60460A	619,254,463	617,788,312	99.76%	91,742,877,201	42.89%	33.263X	DNBSEQ-T7
60465A	626,873,295	625,553,125	99.79%	92,996,387,438	43.22%	33.7173X	DNBSEQ-T7
60471A	602,815,487	601,142,070	99.72%	89,279,458,722	43.01%	32.37X	DNBSEQ-T7
70036A	702,492,125	700,664,408	99.74%	103,871,655,056	42.92%	37.6603X	DNBSEQ-T7
70244A	853,022,329	850,776,880	99.74%	125,876,115,365	43.07%	45.6384X	DNBSEQ-T7
70245A	848,424,335	846,542,607	99.78%	125,554,950,646	43.05%	45.5221X	DNBSEQ-T7
70259A	766,212,847	763,759,774	99.68%	113,347,009,093	43.03%	41.096X	DNBSEQ-T7
70334A	775,888,289	773,820,733	99.73%	114,473,506,668	43.02%	41.504X	DNBSEQ-T7
70522A	768,243,655	766,174,840	99.73%	113,796,825,084	42.69%	41.259X	DNBSEQ-T7
70648A	786,254,660	784,517,890	99.78%	116,571,937,758	42.74%	42.2654X	DNBSEQ-T7
800369B	825,603,419	823,727,320	99.77%	122,417,060,472	42.65%	44.3847X	DNBSEQ-T7
800412A	817,373,644	815,374,714	99.76%	120,941,038,130	42.71%	43.8497X	DNBSEQ-T7
800449A	783,273,689	780,871,894	99.69%	115,959,027,545	42.73%	42.0431X	DNBSEQ-T7
L202680B	674,215,025	672,695,738	99.77%	99,908,690,415	42.53%	36.2238X	DNBSEQ-T7
L212695A	784,538,078	782,266,206	99.71%	116,117,487,162	43.14%	42.1003X	DNBSEQ-T7
L212859A	808,014,502	805,991,369	99.75%	119,763,635,795	43.07%	43.4223X	DNBSEQ-T7
L212991A	610,364,989	608,708,370	99.73%	90,369,696,390	42.88%	32.7652X	DNBSEQ-T7
L800058A	646,762,345	644,747,043	99.69%	95,811,862,410	42.92%	34.7384X	DNBSEQ-T7
L800733A	764,837,205	762,734,996	99.73%	113,331,230,488	42.90%	41.0902X	DNBSEQ-T7
SAMN05216027	215,951,725	215,543,910	99.81%	29,438,492,100	41.09%	10.6727X	NextSeq 550
SAMN05216028	195,943,023	195,676,473	99.86%	27,626,013,432	41.59%	10.0156X	NextSeq 550
SAMN05216029	219,949,175	219,621,692	99.85%	31,459,805,231	41.58%	11.4056X	NextSeq 550
SAMN05216030	276,377,779	276,134,880	99.91%	40,334,924,795	42.54%	14.6233X	NextSeq 550

Sample ID	Total reads	Mapped reads	Mapping rate	Total sequenced bases (bp)	GC content	Mean depth	Instrument
SAMN05216031	225,023,459	224,759,974	99.88%	32,222,628,021	42.89%	11.6822X	NextSeq 550
SAMN05216032	193,063,018	192,827,157	99.88%	28,029,562,847	41.77%	10.1621X	NextSeq 550
SAMN05788541	476,435,041	475,412,782	99.79%	47,103,329,119	41.27%	17.076X	Illumina HiSeq 2000、Illumina HiSeq 2500
SAMN05788542	579,814,871	578,518,524	99.78%	57,243,788,794	41.94%	20.7527X	Illumina HiSeq 2000、Illumina HiSeq 2500
SAMN05788543	472,802,309	472,209,612	99.87%	46,767,210,425	41.21%	16.9542X	Illumina HiSeq 2000、Illumina HiSeq 2500
SAMN05788544	468,061,675	452,380,069	96.65%	44,801,876,831	40.99%	16.2416X	Illumina HiSeq 2000
SAMN05788545	630,530,972	611,725,710	97.02%	60,646,691,161	41.58%	21.986X	Illumina HiSeq 2000、Illumina HiSeq 2500
SAMN05788546	460,340,810	451,943,588	98.18%	44,775,441,773	40.91%	16.232X	Illumina HiSeq 2000
SAMN09379696	229,102,315	225,313,850	98.35%	32,116,048,786	42.63%	11.6446X	HiSeq X Ten
SAMN09379698	224,358,678	219,615,618	97.89%	31,024,957,849	43.07%	11.2487X	HiSeq X Ten
SAMN09379706	227,895,533	215,271,680	94.46%	30,227,834,439	43.58%	10.9599X	HiSeq X Ten
SAMN09379708	366,309,615	362,574,861	98.98%	51,386,145,398	43.34%	18.6313X	HiSeq X Ten
SAMN09379717	275,092,612	269,763,071	98.06%	38,070,658,409	44.07%	13.8033X	HiSeq X Ten
SAMN09379718	252,896,584	248,397,043	98.22%	35,141,693,156	43.72%	12.7415X	HiSeq X Ten
SAMN09379730	317,220,441	311,709,843	98.26%	44,009,749,405	43.49%	15.9566X	HiSeq X Ten
SAMN09379731	275,642,741	265,978,115	96.49%	37,439,242,585	43.45%	13.5744X	HiSeq X Ten
SAMN09379734	318,455,086	312,575,341	98.15%	44,413,178,859	43.60%	16.103X	HiSeq X Ten
SAMN09379767	236,836,147	230,631,872	97.38%	32,827,672,339	42.86%	11.9026X	HiSeq X Ten
SAMN09379768	272,687,322	265,504,231	97.37%	37,414,810,093	43.62%	13.5656X	HiSeq X Ten
SAMN09379772	233,294,027	203,243,246	87.12%	28,696,422,273	43.72%	10.4045X	HiSeq X Ten
SAMN09379777	290,628,439	235,382,217	80.99%	33,159,237,760	44.24%	12.0229X	HiSeq X Ten
SAMN09379778	245,984,593	188,218,391	76.52%	26,189,407,462	45.54%	9.4959X	HiSeq X Ten
SAMN09379782	275,239,343	247,647,059	89.98%	34,837,637,640	44.52%	12.6313X	HiSeq X Ten

Sample ID	Total reads	Mapped reads	Mapping rate	Total sequenced bases (bp)	GC content	Mean depth	Instrument
SAMN09379785	407,007,271	365,758,041	89.87%	51,806,548,162	43.78%	18.7839X	HiSeq X Ten
SAMN09379786	244,029,005	209,575,525	85.88%	29,392,211,514	43.99%	10.657X	HiSeq X Ten
SAMN09379807	271,533,176	262,549,196	96.69%	36,862,632,760	44.16%	13.3656X	HiSeq X Ten
SAMN09379809	193,992,567	191,134,187	98.53%	26,922,831,428	43.69%	9.7614X	HiSeq X Ten
SAMN09379822	406,476,319	402,795,834	99.09%	57,233,225,667	43.95%	20.751X	HiSeq X Ten
SAMN09379823	284,977,766	273,366,046	95.93%	38,563,249,271	44.57%	13.9821X	HiSeq X Ten
SAMN09379824	339,115,564	332,105,076	97.93%	46,743,039,047	44.23%	16.9481X	HiSeq X Ten
SAMN09379825	289,305,641	277,912,017	96.06%	39,046,218,990	44.62%	14.1573X	HiSeq X Ten
SAMN09379827	338,593,697	319,417,421	94.34%	44,646,647,064	45.37%	16.1879X	HiSeq X Ten
SAMN09379828	228,930,553	220,827,182	96.46%	31,107,357,801	44.01%	11.2785X	HiSeq X Ten
SAMN09379831	255,448,418	252,383,590	98.80%	35,656,509,205	44.08%	12.9283X	HiSeq X Ten
SAMN09379832	213,602,216	210,271,222	98.44%	29,475,460,614	44.17%	10.6871X	HiSeq X Ten
SAMN10182839	326,698,859	316,618,624	96.91%	31,502,783,172	40.74%	11.4206X	Illumina HiSeq 2000
SAMN10182840	355,911,394	345,889,859	97.18%	34,378,201,243	40.78%	12.463X	Illumina HiSeq 2000
SAMN10182841	267,590,267	263,249,259	98.38%	26,067,089,996	40.45%	9.4499X	Illumina HiSeq 2000
SAMN10182842	343,696,689	322,733,164	93.90%	32,078,449,882	41.43%	11.6292X	Illumina HiSeq 2000
SAMN10940558	452,227,109	434,964,827	96.18%	43,044,818,835	41.73%	15.6047X	Illumina HiSeq 2000
SAMN10940559	441,044,157	426,770,142	96.76%	42,208,078,440	41.52%	15.3013X	Illumina HiSeq 2000
SAMN10940560	450,831,116	449,011,307	99.60%	44,487,190,028	41.47%	16.1275X	Illumina HiSeq 2000、Illumina HiSeq 2500
SAMN10940561	492,644,675	482,450,311	97.93%	47,844,392,795	41.78%	17.3446X	Illumina HiSeq 2000、Illumina HiSeq 2500
SAMN10940562	385,770,607	385,312,688	99.88%	38,140,673,932	41.81%	13.8269X	Illumina HiSeq 2000
SRR1262805	235,210,714	233,640,006	99.33%	22,994,192,749	43.85%	8.3363X	Illumina HiSeq 2000
SRR1262843	286,602,892	270,443,098	94.36%	26,565,804,890	44.19%	9.6317X	Illumina HiSeq 2000

Sample ID	Total reads	Mapped reads	Mapping rate	Total sequenced bases (bp)	GC content	Mean depth	Instrument
SRR1525617	237,637,014	236,541,305	99.54%	23,466,098,290	40.60%	8.5074X	Illumina HiSeq 2000
SRR1525618	276,926,139	274,479,568	99.12%	27,308,541,209	41.64%	9.9X	Illumina HiSeq 2000
SRR1525619	243,961,094	242,267,919	99.31%	24,054,189,648	40.87%	8.7202X	Illumina HiSeq 2000
SRR1525620	281,551,093	279,785,253	99.37%	27,810,455,128	41.29%	10.0819X	Illumina HiSeq 2000
SRR1525621	256,508,162	254,224,695	99.11%	25,285,029,732	41.60%	9.1665X	Illumina HiSeq 2000
SRR1525622	258,235,576	256,373,429	99.28%	25,464,160,785	41.21%	9.2314X	Illumina HiSeq 2000
SRR1525623	247,393,027	245,892,429	99.39%	24,436,861,377	41.30%	8.8589X	Illumina HiSeq 2000
SRR1525624	294,106,494	293,730,373	99.87%	29,234,285,573	42.53%	10.5982X	Illumina HiSeq 2000
SRR1525625	249,857,124	247,371,318	99.01%	24,596,787,590	41.65%	8.9169X	Illumina HiSeq 2000
SRR1525626	297,198,138	294,363,024	99.05%	29,294,298,627	41.97%	10.6199X	Illumina HiSeq 2000
SRR1525627	228,031,510	226,696,550	99.41%	22,526,334,790	40.52%	8.1663X	Illumina HiSeq 2000
SRR1525700	280,266,779	278,353,511	99.32%	27,687,589,359	41.14%	10.0373X	Illumina HiSeq 2000
SRR1525701	231,382,525	229,494,052	99.18%	22,512,564,012	41.03%	8.1613X	Illumina HiSeq 2000
SRR1525702	315,950,704	314,002,395	99.38%	31,266,834,580	41.51%	11.335X	Illumina HiSeq 2000
SRR1525703	338,755,659	323,849,737	95.60%	32,075,607,297	41.20%	11.6282X	Illumina HiSeq 2000
SRR1525704	255,991,538	253,693,390	99.10%	25,211,725,146	41.68%	9.1399X	Illumina HiSeq 2000
SRR1525705	265,659,690	263,294,154	99.11%	26,217,357,243	41.48%	9.5044X	Illumina HiSeq 2000
SRR1525706	280,926,938	279,241,457	99.40%	27,772,090,457	41.16%	10.068X	Illumina HiSeq 2000
SRR1525707	249,855,452	247,997,477	99.26%	24,659,096,393	41.35%	8.9395X	Illumina HiSeq 2000
SRR1525708	305,180,668	303,042,884	99.30%	30,177,908,609	41.84%	10.9402X	Illumina HiSeq 2000
SRR1525709	267,218,516	265,580,952	99.39%	26,366,173,402	41.07%	9.5583X	Illumina HiSeq 2000
SRR1525712	232,678,561	231,309,540	99.41%	22,979,925,669	41.47%	8.3307X	Illumina HiSeq 2000
SRR1525713	255,894,104	253,690,711	99.14%	25,218,509,559	41.66%	9.1423X	Illumina HiSeq 2000

Sample ID	Total reads	Mapped reads	Mapping rate	Total sequenced bases (bp)	GC content	Mean depth	Instrument
SRR1525714	248,311,918	244,414,401	98.43%	24,302,851,346	41.47%	8.8104X	Illumina HiSeq 2000
SRR1525715	303,763,395	301,684,904	99.32%	30,041,236,781	41.52%	10.8908X	Illumina HiSeq 2000
SRR1525716	282,820,241	280,913,054	99.33%	27,937,931,367	41.36%	10.1281X	Illumina HiSeq 2000
SRR1525717	284,845,424	280,598,770	98.51%	27,909,509,498	41.36%	10.1178X	Illumina HiSeq 2000
SRR24013030	537,788,714	537,092,014	99.87%	53,548,337,306	42.08%	19.4132X	Illumina HiSeq 2000
SRR24013031	700,052,591	699,072,125	99.86%	69,644,959,734	43.42%	25.2487X	Illumina HiSeq 2000
SRR24013032	700,188,805	699,329,320	99.88%	69,712,682,794	42.77%	25.2732X	Illumina HiSeq 2000
SRR24013033	685,987,628	685,210,407	99.89%	68,241,555,593	42.43%	24.7401X	Illumina HiSeq 2000
SRR24013034	703,855,878	703,228,590	99.91%	69,990,932,104	42.63%	25.3743X	Illumina HiSeq 2000
SRR24013035	690,674,304	689,787,945	99.87%	68,739,462,656	42.12%	24.9205X	Illumina HiSeq 2000
SRR24013036	683,519,686	682,690,887	99.88%	68,006,947,980	43.47%	24.6549X	Illumina HiSeq 2000
SRR24013037	607,489,201	606,894,153	99.90%	60,178,345,981	44.01%	21.8166X	Illumina HiSeq 2000
SRR24013038	649,884,125	649,192,442	99.89%	64,644,282,577	43.44%	23.4356X	Illumina HiSeq 2000
SRR24013039	690,706,250	688,734,535	99.71%	68,638,333,733	43.04%	24.8837X	Illumina HiSeq 2000
SRR24013040	677,708,284	676,764,012	99.86%	67,427,744,763	42.92%	24.445X	Illumina HiSeq 2000
SRR24013041	647,769,595	646,952,815	99.87%	64,430,370,375	44.54%	23.358X	Illumina HiSeq 2000
SRR24013042	674,021,066	673,167,400	99.87%	67,086,920,569	42.43%	24.3214X	Illumina HiSeq 2000
SRR24013043	683,547,916	682,505,361	99.85%	67,953,319,527	43.18%	24.6355X	Illumina HiSeq 2000
SRR24013044	672,545,914	671,537,542	99.85%	66,896,414,763	43.34%	24.2523X	Illumina HiSeq 2000
SRR24013045	685,101,917	684,261,785	99.88%	68,120,946,393	43.39%	24.6963X	Illumina HiSeq 2000
SRR24013046	663,376,583	662,468,317	99.86%	65,986,481,619	43.08%	23.9225X	Illumina HiSeq 2000
SRR24013047	391,352,621	390,861,351	99.87%	38,891,858,371	43.95%	14.0994X	Illumina HiSeq 2000
SRR24013048	660,662,407	659,944,278	99.89%	65,686,469,442	44.56%	23.8135X	Illumina HiSeq 2000

Sample ID	Total reads	Mapped reads	Mapping rate	Total sequenced bases (bp)	GC content	Mean depth	Instrument
SRR24013049	611,243,931	610,415,957	99.86%	60,772,614,205	43.17%	22.0321X	Illumina HiSeq 2000
SRR24013050	592,146,746	591,174,532	99.84%	58,828,641,053	43.95%	21.3273X	Illumina HiSeq 2000
SRR24013052	662,456,608	661,635,309	99.88%	65,919,481,421	43.29%	23.8981X	Illumina HiSeq 2000
SRR24013053	661,736,957	660,818,579	99.86%	65,734,829,339	44.31%	23.8311X	Illumina HiSeq 2000
SRR24013054	656,268,193	655,431,146	99.87%	65,177,318,508	44.08%	23.6291X	Illumina HiSeq 2000
SRR24013058	595,111,381	593,974,932	99.81%	59,151,867,789	43.82%	21.4444X	Illumina HiSeq 2000
SRR24013059	687,611,990	686,696,615	99.87%	68,426,866,319	42.90%	24.8071X	Illumina HiSeq 2000
SRR30016219	199,791,812	199,482,038	99.84%	29,409,196,895	43.44%	10.6624X	BGISEQ-50
SRR30016220	198,014,593	197,635,548	99.81%	29,199,157,991	43.19%	10.5863X	BGISEQ-50
SRR30016221	188,970,360	188,669,556	99.84%	27,970,195,400	43.76%	10.1409X	BGISEQ-50
SRR30016222	202,982,179	202,472,017	99.75%	29,977,563,220	43.23%	10.8685X	BGISEQ-50
SRR30016223	201,223,871	200,906,880	99.84%	29,719,602,613	43.92%	10.7751X	BGISEQ-50
SRR30016225	223,685,424	223,358,829	99.85%	32,989,901,926	43.50%	11.9607X	BGISEQ-50
SRR30016226	216,045,909	215,617,752	99.80%	31,832,538,030	43.24%	11.5411X	BGISEQ-50
SRR30016228	200,010,196	199,506,183	99.75%	29,489,537,327	43.11%	10.6915X	BGISEQ-50
SRR30016240	205,909,818	205,572,797	99.84%	30,370,170,010	43.52%	11.0109X	BGISEQ-50
SRR30016241	213,365,234	212,910,791	99.79%	31,461,058,631	43.35%	11.4063X	BGISEQ-50
SRR30016242	243,401,394	242,978,538	99.83%	35,924,292,648	43.13%	13.0245X	BGISEQ-50
SRR30016244	211,856,780	211,578,915	99.87%	31,255,342,545	43.56%	11.3318X	BGISEQ-50
SRR30016245	206,079,549	205,739,263	99.83%	30,404,925,436	43.68%	11.0234X	BGISEQ-50
SRR30016246	206,870,249	206,585,973	99.86%	30,529,070,361	43.37%	11.0686X	BGISEQ-50
SRR30016247	207,245,266	206,920,777	99.84%	30,593,995,739	43.53%	11.092X	BGISEQ-50
SRR30016248	193,704,244	193,430,711	99.86%	28,607,789,952	43.33%	10.372X	BGISEQ-50

Sample ID	Total reads	Mapped reads	Mapping rate	Total sequenced bases (bp)	GC content	Mean depth	Instrument
SRR30016249	204,235,513	203,948,033	99.86%	30,167,759,138	43.15%	10.9378X	BGISEQ-50
SRR30016250	194,953,086	194,667,614	99.85%	28,778,791,062	43.19%	10.434X	BGISEQ-50
SRR33223349	217,323,227	217,131,943	99.91%	32,367,790,734	43.78%	11.7354X	DNBSEQ-T7
SRR33223350	217,822,125	217,611,029	99.90%	32,430,629,182	43.58%	11.7582X	DNBSEQ-T7
SRR33223351	217,970,579	217,780,637	99.91%	32,447,915,127	43.61%	11.7645X	DNBSEQ-T7
SRR33223352	217,521,422	217,240,443	99.87%	32,357,713,506	43.55%	11.7317X	DNBSEQ-T7
SRR33223353	217,412,995	217,229,694	99.92%	32,366,128,475	43.66%	11.7349X	DNBSEQ-T7
SRR33223354	191,790,814	191,529,639	99.86%	28,552,018,251	43.59%	10.3519X	DNBSEQ-T7
SRR33223355	217,722,300	217,523,758	99.91%	32,422,760,235	43.61%	11.7554X	DNBSEQ-T7
SRR33223356	216,372,968	216,180,778	99.91%	32,238,138,224	43.68%	11.6883X	DNBSEQ-T7
SRR33223357	208,628,562	208,443,782	99.91%	31,067,376,077	43.73%	11.264X	DNBSEQ-T7
SRR33223358	217,844,986	217,614,673	99.89%	32,423,275,849	43.66%	11.7555X	DNBSEQ-T7
SRR33223359	188,208,088	188,019,322	99.90%	28,012,277,230	43.74%	10.1562X	DNBSEQ-T7
SRR33223360	217,741,786	217,486,051	99.88%	32,410,740,367	43.60%	11.751X	DNBSEQ-T7
SRR33223361	215,974,242	215,859,693	99.95%	32,158,642,130	44.58%	11.6595X	DNBSEQ-T7
SRR33223362	213,172,150	212,913,211	99.88%	31,716,563,935	43.35%	11.4993X	DNBSEQ-T7
SRR33223363	218,192,260	217,990,082	99.91%	32,474,572,180	43.54%	11.7741X	DNBSEQ-T7
SRR33223364	218,025,917	217,834,015	99.91%	32,485,688,078	43.74%	11.7781X	DNBSEQ-T7
SRR33223365	205,588,967	205,388,416	99.90%	30,638,926,587	43.72%	11.1086X	DNBSEQ-T7
SRR33223366	217,914,150	217,687,813	99.90%	32,470,745,375	43.57%	11.7727X	DNBSEQ-T7
SRR33223369	217,870,039	217,593,813	99.87%	32,446,412,954	43.82%	11.7639X	DNBSEQ-T7
SRR33223370	217,675,018	217,390,197	99.87%	32,412,494,560	43.83%	11.7516X	DNBSEQ-T7
SRR33223371	217,902,248	216,967,869	99.57%	32,323,543,665	43.75%	11.7194X	DNBSEQ-T7

Sample ID	Total reads	Mapped reads	Mapping rate	Total sequenced bases (bp)	GC content	Mean depth	Instrument
SRR33223373	217,567,233	217,306,832	99.88%	32,408,933,187	43.84%	11.7503X	DNBSEQ-T7
SRR33223374	217,606,059	217,081,361	99.76%	32,325,682,917	43.85%	11.7203X	DNBSEQ-T7
SRR33223375	217,737,844	217,521,063	99.90%	32,438,046,854	43.77%	11.7608X	DNBSEQ-T7
SRR33223376	217,835,817	217,416,887	99.81%	32,414,912,406	43.76%	11.7524X	DNBSEQ-T7
SRR33223377	218,070,690	217,834,115	99.89%	32,481,441,943	43.69%	11.7766X	DNBSEQ-T7
SRR33223378	217,849,859	217,550,941	99.86%	32,446,531,523	43.75%	11.764X	DNBSEQ-T7
SRR33223379	218,209,431	218,010,124	99.91%	32,498,572,462	43.57%	11.7828X	DNBSEQ-T7
SRR33223380	218,258,813	218,042,651	99.90%	32,525,454,488	43.58%	11.7925X	DNBSEQ-T7
SRR33223381	217,916,351	217,632,736	99.87%	32,443,719,856	43.72%	11.763X	DNBSEQ-T7
SRR33223383	217,958,317	217,398,872	99.74%	32,407,583,359	43.69%	11.7499X	DNBSEQ-T7
SRR33223384	217,771,990	217,514,265	99.88%	32,439,843,882	43.66%	11.7616X	DNBSEQ-T7
SRR33223385	218,044,430	217,823,683	99.90%	32,478,251,725	43.70%	11.7754X	DNBSEQ-T7
SRR33223386	217,874,830	217,527,248	99.84%	32,423,875,054	43.70%	11.7557X	DNBSEQ-T7
SRR33223387	217,677,740	217,313,434	99.83%	32,394,419,972	43.75%	11.7452X	DNBSEQ-T7
SRR33223388	218,149,545	217,887,053	99.88%	32,488,886,049	43.46%	11.7793X	DNBSEQ-T7
SRR33223390	217,788,142	217,508,524	99.87%	32,431,786,363	43.81%	11.7586X	DNBSEQ-T7
SRR33223391	218,190,131	217,916,597	99.87%	32,493,755,560	43.59%	11.7811X	DNBSEQ-T7
SRR33223392	217,971,496	217,656,203	99.86%	32,433,579,633	43.53%	11.7593X	DNBSEQ-T7
SRR33223393	217,816,156	217,632,012	99.92%	32,444,849,371	43.66%	11.7634X	DNBSEQ-T7
SRR33223394	217,659,996	217,431,117	99.89%	32,426,168,644	43.76%	11.7565X	DNBSEQ-T7
SRR33223395	214,417,950	214,139,403	99.87%	31,932,797,530	43.65%	11.5777X	DNBSEQ-T7
SRR33223396	217,997,167	217,517,401	99.78%	32,434,765,109	43.71%	11.7597X	DNBSEQ-T7
SRR33223397	217,776,304	217,340,879	99.80%	32,402,402,132	43.75%	11.7479X	DNBSEQ-T7

Sample ID	Total reads	Mapped reads	Mapping rate	Total sequenced bases (bp)	GC content	Mean depth	Instrument
SRR33223398	218,060,224	217,694,970	99.83%	32,447,067,118	43.67%	11.7642X	DNBSEQ-T7
SRR33223399	218,162,710	217,946,933	99.90%	32,496,793,003	43.61%	11.7822X	DNBSEQ-T7
SRR33223400	217,489,571	217,284,321	99.91%	32,374,460,622	43.98%	11.7379X	DNBSEQ-T7
SRR33223401	217,847,923	217,645,307	99.91%	32,453,605,347	43.84%	11.7664X	DNBSEQ-T7
SRR33223402	217,754,421	217,564,657	99.91%	32,446,197,433	43.72%	11.7638X	DNBSEQ-T7
SRR33223403	218,537,602	218,250,512	99.87%	32,545,269,012	43.43%	11.7998X	DNBSEQ-T7
SRR33223404	217,620,186	217,376,607	99.89%	32,404,038,226	43.65%	11.7485X	DNBSEQ-T7
SRR33223405	217,797,971	217,589,650	99.90%	32,447,849,118	43.86%	11.7644X	DNBSEQ-T7
SRR33223406	217,844,618	217,647,697	99.91%	32,471,515,782	43.76%	11.773X	DNBSEQ-T7
SRR33223407	200,013,972	199,837,843	99.91%	29,816,784,919	44.58%	10.8104X	DNBSEQ-T7
SRR33223408	217,898,785	217,651,002	99.89%	32,452,489,402	43.70%	11.7662X	DNBSEQ-T7
SRR33223409	217,580,320	217,380,948	99.91%	32,432,138,450	43.87%	11.7588X	DNBSEQ-T7
SRR33223410	217,654,961	217,435,130	99.90%	32,431,761,580	43.90%	11.7586X	DNBSEQ-T7
SRR33223411	218,022,095	217,841,404	99.92%	32,484,535,087	43.80%	11.7777X	DNBSEQ-T7
SRR33223412	217,767,996	217,592,413	99.92%	32,459,880,602	43.80%	11.7687X	DNBSEQ-T7
SRR33223413	217,735,650	217,541,198	99.91%	32,455,755,092	43.81%	11.7673X	DNBSEQ-T7
SRR33223414	210,272,631	210,039,744	99.89%	31,318,769,954	43.99%	11.355X	DNBSEQ-T7
SRR33223415	218,255,448	218,051,513	99.91%	32,484,196,019	43.43%	11.7776X	DNBSEQ-T7
SRR33223416	207,545,785	207,327,557	99.89%	30,932,769,777	43.80%	11.215X	DNBSEQ-T7
SRR33223417	209,612,653	209,398,401	99.90%	31,229,642,380	43.74%	11.3228X	DNBSEQ-T7
SRR33223418	218,017,193	217,843,833	99.92%	32,486,979,159	43.79%	11.7786X	DNBSEQ-T7
SRR33223419	217,767,380	217,189,646	99.73%	32,365,930,825	43.68%	11.7347X	DNBSEQ-T7
SRR33223420	217,888,492	217,654,357	99.89%	32,465,560,825	43.83%	11.7708X	DNBSEQ-T7

Sample ID	Total reads	Mapped reads	Mapping rate	Total sequenced bases (bp)	GC content	Mean depth	Instrument
SRR33223421	218,186,288	217,756,792	99.80%	32,443,584,688	43.53%	11.763X	DNBSEQ-T7
SRR33223422	200,227,208	200,009,320	99.89%	29,834,603,111	43.83%	10.8169X	DNBSEQ-T7
SRR33223423	217,406,945	217,160,235	99.89%	32,359,326,184	43.79%	11.7323X	DNBSEQ-T7
SRR33223424	217,989,184	217,800,638	99.91%	32,487,880,167	43.69%	11.779X	DNBSEQ-T7
SRR33223425	217,672,748	217,430,196	99.89%	32,421,955,193	43.81%	11.755X	DNBSEQ-T7
SRR33223426	218,159,027	217,276,101	99.60%	32,352,519,598	43.41%	11.7298X	DNBSEQ-T7
SRR33223427	217,880,156	217,671,794	99.90%	32,440,115,424	43.68%	11.7617X	DNBSEQ-T7
SRR33223428	217,550,238	217,298,247	99.88%	32,408,346,882	43.82%	11.7501X	DNBSEQ-T7
SRR33223429	217,810,700	217,610,662	99.91%	32,432,198,799	43.59%	11.7587X	DNBSEQ-T7
SRR33223430	217,453,765	217,209,664	99.89%	32,393,741,546	43.96%	11.7448X	DNBSEQ-T7
SRR33223431	217,766,758	217,562,611	99.91%	32,434,838,916	43.59%	11.7596X	DNBSEQ-T7
SRR33223432	217,931,865	217,687,385	99.89%	32,442,739,941	43.59%	11.7626X	DNBSEQ-T7
SRR33223433	217,600,541	217,377,134	99.90%	32,414,848,192	43.88%	11.7525X	DNBSEQ-T7
SRR33223434	217,580,116	217,377,625	99.91%	32,391,129,462	43.83%	11.7438X	DNBSEQ-T7
SRR33223435	217,794,822	217,585,661	99.90%	32,453,531,632	43.69%	11.7665X	DNBSEQ-T7
SRR33223436	217,860,940	217,623,934	99.89%	32,427,665,035	43.46%	11.7571X	DNBSEQ-T7
SRR33223437	217,931,059	217,680,041	99.88%	32,434,498,852	43.52%	11.7596X	DNBSEQ-T7
SRR33223438	217,616,085	217,260,164	99.84%	32,386,586,188	43.71%	11.7422X	DNBSEQ-T7

Table S3. Cross-validation errors for each K value in Simmental cattle populations from different regions

K value	CV error
2	0.26858
3	0.43380
4	0.26792
5	0.26768
6	0.26864
7	0.26960
8	0.27082
9	0.27208
10	0.27353

Table S4. 286 SNP loci significantly associated with twinning trait in Chinese Simmental cattle from Xinjiang region

Chr	Variant ID	Position (bp)	Ref	Alt	Region type	Genes	Allele frequency
1	1:19807728	19807728	G	A	intergenic	CXADR, MIR125B-2	0.0877193
1	1:19817312	19817312	T	C	intergenic	CXADR, MIR125B-2	0.0877193
1	1:19857358	19857358	G	T	intergenic	CXADR, MIR125B-2	0.0877193
1	1:19859704	19859704	C	T	intergenic	CXADR, MIR125B-2	0.0877193
1	1:33419929	33419929	G	T	intronic	CADM2	0.0877193
1	1:67357624	67357624	G	C	intronic	SEMA5B	0.0877193
1	1:77557754	77557754	A	G	intronic	TP63	0.0877193
1	1:77558635	77558635	A	G	intronic	TP63	0.0877193
1	1:77559057	77559057	C	T	intronic	TP63	0.0877193
1	1:77575170	77575170	A	G	intronic	TP63	0.0982143
1	1:77727027	77727027	T	A	intergenic	TP63, TPRG1	0.0892857
1	1:77737822	77737822	G	A	intergenic	TP63, TPRG1	0.0877193
1	1:77744666	77744666	G	C	intergenic	TP63, TPRG1	0.0877193
1	1:77967572	77967572	T	C	intergenic	TP63, TPRG1	0.0877193
1	1:78041296	78041296	G	T	intronic	TPRG1	0.0877193
1	1:93886002	93886002	T	C	intronic	NLGN1	0.0877193
2	2:32594609	32594609	G	C	intergenic	GRB14, FIGN	0.0877193
2	2:41602501	41602501	G	A	intergenic	KCNJ3, GALNT13	0.0877193
2	2:60733735	60733735	G	A	intergenic	TRNAC-GCA, CXCR4	0.0877193
2	2:60746335	60746335	G	A	intergenic	TRNAC-GCA, CXCR4	0.0877193
3	3:13113421	13113421	G	A	intergenic	LOC784007, LOC112445940	0.0877193
3	3:19902244	19902244	G	A	intergenic	TRNAG-CCC, ARNT	0.0877193
3	3:19935149	19935149	A	G	intronic	ARNT	0.0877193
3	3:19999722	19999722	C	T	intronic	CTSS	0.0964912

Chr	Variant ID	Position (bp)	Ref	Alt	Region type	Genes	Allele frequency
3	3:20001223	20001223	A	G	intronic	CTSS	0.0964912
3	3:20001657	20001657	A	T	intronic	CTSS	0.0877193
3	3:20002019	20002019	A	G	intronic	CTSS	0.0964912
3	3:20002285	20002285	T	C	intronic	CTSS	0.0964912
3	3:20002695	20002695	G	A	intronic	CTSS	0.0877193
3	3:20002781	20002781	C	T	intronic	CTSS	0.0964912
3	3:20004450	20004450	T	C	UTR3	CTSS	0.0964912
3	3:20006021	20006021	G	A	intergenic	CTSS, HORMAD1	0.0982143
3	3:20006643	20006643	G	A	intergenic	CTSS, HORMAD1	0.0964912
3	3:20006955	20006955	C	T	intergenic	CTSS, HORMAD1	0.0964912
3	3:20007496	20007496	T	C	intergenic	CTSS, HORMAD1	0.0964912
3	3:20007541	20007541	G	A	intergenic	CTSS, HORMAD1	0.0877193
3	3:20008260	20008260	C	G	intergenic	CTSS, HORMAD1	0.0964912
3	3:20008352	20008352	G	C	intergenic	CTSS, HORMAD1	0.0877193
3	3:20009207	20009207	T	C	intergenic	CTSS, HORMAD1	0.0877193
3	3:20010186	20010186	C	T	intergenic	CTSS, HORMAD1	0.0964912
3	3:20010894	20010894	G	A	intergenic	CTSS, HORMAD1	0.0877193
3	3:20010926	20010926	C	T	intergenic	CTSS, HORMAD1	0.0877193
3	3:20010960	20010960	G	A	intergenic	CTSS, HORMAD1	0.0964912
3	3:20011012	20011012	A	G	intergenic	CTSS, HORMAD1	0.0964912
3	3:20011035	20011035	C	T	intergenic	CTSS, HORMAD1	0.0964912
3	3:20011413	20011413	A	G	intergenic	CTSS, HORMAD1	0.0964912
3	3:20011426	20011426	G	C	intergenic	CTSS, HORMAD1	0.0964912
3	3:20012153	20012153	T	C	intergenic	CTSS, HORMAD1	0.0964912
3	3:20015196	20015196	G	A	intergenic	CTSS, HORMAD1	0.0964912

Chr	Variant ID	Position (bp)	Ref	Alt	Region type	Genes	Allele frequency
3	3:20018475	20018475	G	T	intronic	HORMAD1	0.0982143
3	3:20020067	20020067	C	T	intronic	HORMAD1	0.0964912
3	3:20020145	20020145	T	G	intronic	HORMAD1	0.0877193
3	3:20022666	20022666	C	T	intronic	HORMAD1	0.0877193
3	3:20022817	20022817	G	T	intronic	HORMAD1	0.0877193
3	3:20023189	20023189	A	G	intronic	HORMAD1	0.0964912
3	3:20023194	20023194	T	A	intronic	HORMAD1	0.0964912
3	3:20024918	20024918	T	C	intronic	HORMAD1	0.0964912
3	3:20024954	20024954	T	G	intronic	HORMAD1	0.0964912
3	3:20025156	20025156	A	G	intronic	HORMAD1	0.0964912
3	3:20025487	20025487	G	C	intronic	HORMAD1	0.0964912
3	3:20025739	20025739	C	A	intronic	HORMAD1	0.0877193
3	3:20026543	20026543	A	G	intronic	HORMAD1	0.0964912
3	3:20026940	20026940	A	G	intronic	HORMAD1	0.0982143
3	3:20027337	20027337	G	A	intronic	HORMAD1	0.0877193
3	3:20027383	20027383	G	A	intronic	HORMAD1	0.0877193
3	3:20027691	20027691	A	G	intronic	HORMAD1	0.0964912
3	3:20029358	20029358	T	C	intronic	HORMAD1	0.0877193
3	3:20029932	20029932	T	C	intronic	HORMAD1	0.0964912
3	3:20030293	20030293	T	G	intronic	HORMAD1	0.0877193
3	3:20030812	20030812	C	T	intronic	HORMAD1	0.0892857
3	3:20031386	20031386	C	G	intronic	HORMAD1	0.0964912
3	3:20032664	20032664	A	G	intronic	HORMAD1	0.0964912
3	3:20034510	20034510	C	T	intronic	HORMAD1	0.0877193
3	3:20038518	20038518	G	A	intronic	GOLPH3L	0.0892857

Chr	Variant ID	Position (bp)	Ref	Alt	Region type	Genes	Allele frequency
3	3:20040221	20040221	T	C	intronic	GOLPH3L	0.0877193
3	3:20040502	20040502	T	C	intronic	GOLPH3L	0.0964912
3	3:20041813	20041813	G	T	intronic	GOLPH3L	0.0964912
3	3:20042268	20042268	G	T	intronic	GOLPH3L	0.0964912
3	3:20044261	20044261	G	A	intronic	GOLPH3L	0.0877193
3	3:20044488	20044488	A	G	intronic	GOLPH3L	0.0877193
3	3:20045589	20045589	C	T	intronic	GOLPH3L	0.0964912
3	3:20048795	20048795	T	G	intronic	GOLPH3L	0.0892857
3	3:20048928	20048928	A	C	intronic	GOLPH3L	0.0964912
3	3:20048954	20048954	C	G	intronic	GOLPH3L	0.0877193
3	3:20049009	20049009	T	C	intronic	GOLPH3L	0.0964912
3	3:20049011	20049011	A	T	intronic	GOLPH3L	0.0964912
3	3:20049677	20049677	G	T	intronic	GOLPH3L	0.0982143
3	3:20050090	20050090	T	C	intronic	GOLPH3L	0.1
3	3:20050194	20050194	T	C	intronic	GOLPH3L	0.0964912
3	3:20050853	20050853	G	T	intronic	GOLPH3L	0.0982143
3	3:20050912	20050912	A	G	intronic	GOLPH3L	0.0982143
3	3:20051140	20051140	C	A	intronic	GOLPH3L	0.0877193
3	3:20051303	20051303	G	C	intronic	GOLPH3L	0.0964912
3	3:20051504	20051504	T	G	intronic	GOLPH3L	0.0964912
3	3:20051633	20051633	T	G	intronic	GOLPH3L	0.0877193
3	3:20052818	20052818	G	A	intronic	GOLPH3L	0.0982143
3	3:20053252	20053252	A	G	intronic	GOLPH3L	0.0964912
3	3:20053703	20053703	C	T	intronic	GOLPH3L	0.0877193
3	3:20054238	20054238	T	C	intronic	GOLPH3L	0.0877193

Chr	Variant ID	Position (bp)	Ref	Alt	Region type	Genes	Allele frequency
3	3:20054437	20054437	A	T	intronic	GOLPH3L	0.0964912
3	3:20054831	20054831	A	T	intronic	GOLPH3L	0.0982143
3	3:20058629	20058629	C	G	intronic	GOLPH3L	0.0964912
3	3:20058975	20058975	G	A	intronic	GOLPH3L	0.0877193
3	3:20059144	20059144	G	A	intronic	GOLPH3L	0.0964912
3	3:20060337	20060337	T	C	intronic	GOLPH3L	0.0964912
3	3:20060338	20060338	G	A	intronic	GOLPH3L	0.0964912
3	3:20060801	20060801	A	G	intronic	GOLPH3L	0.0892857
3	3:20061307	20061307	A	G	intronic	GOLPH3L	0.0964912
3	3:20061540	20061540	A	G	intronic	GOLPH3L	0.0964912
3	3:20063117	20063117	A	T	intronic	GOLPH3L	0.0964912
3	3:20063537	20063537	C	T	intronic	GOLPH3L	0.0964912
3	3:20064494	20064494	T	G	intronic	GOLPH3L	0.0964912
3	3:20064495	20064495	T	C	intronic	GOLPH3L	0.0964912
3	3:20065779	20065779	A	G	intronic	GOLPH3L	0.0964912
3	3:20066102	20066102	T	C	intronic	GOLPH3L	0.0877193
3	3:20066232	20066232	T	C	intronic	GOLPH3L	0.0877193
3	3:20068261	20068261	G	C	intronic	GOLPH3L	0.0964912
3	3:20068650	20068650	C	T	intronic	GOLPH3L	0.0877193
3	3:20069192	20069192	G	T	intronic	GOLPH3L	0.0982143
3	3:20070710	20070710	G	T	intronic	GOLPH3L	0.0877193
3	3:20071879	20071879	T	G	intronic	GOLPH3L	0.0877193
3	3:20074072	20074072	C	T	intronic	GOLPH3L	0.0964912
3	3:20074446	20074446	A	G	intronic	GOLPH3L	0.0964912
3	3:20077597	20077597	G	A	intronic	GOLPH3L	0.0964912

Chr	Variant ID	Position (bp)	Ref	Alt	Region type	Genes	Allele frequency
3	3:20078246	20078246	A	G	intronic	GOLPH3L	0.0877193
3	3:20080425	20080425	A	T	intronic	GOLPH3L	0.0877193
3	3:20081849	20081849	A	G	intronic	GOLPH3L	0.0964912
3	3:20082409	20082409	A	G	intronic	GOLPH3L	0.0877193
3	3:20083654	20083654	G	C	UTR3	GOLPH3L	0.0877193
3	3:20083958	20083958	C	T	UTR3	GOLPH3L	0.0964912
3	3:20085318	20085318	T	C	downstream	GOLPH3L	0.0964912
3	3:20085547	20085547	C	G	downstream	GOLPH3L	0.0964912
3	3:20086417	20086417	A	G	intergenic	GOLPH3L, ENSA	0.0964912
3	3:20086715	20086715	C	G	intergenic	GOLPH3L, ENSA	0.0877193
3	3:20087583	20087583	G	A	intergenic	GOLPH3L, ENSA	0.0877193
3	3:20087648	20087648	T	G	intergenic	GOLPH3L, ENSA	0.0877193
3	3:20087979	20087979	G	A	intergenic	GOLPH3L, ENSA	0.0877193
3	3:20088607	20088607	A	G	intergenic	GOLPH3L, ENSA	0.0964912
3	3:20088617	20088617	C	T	intergenic	GOLPH3L, ENSA	0.0964912
3	3:20089281	20089281	T	C	intergenic	GOLPH3L, ENSA	0.0964912
3	3:20089406	20089406	T	G	intergenic	GOLPH3L, ENSA	0.0964912
3	3:20095807	20095807	G	A	intergenic	GOLPH3L, ENSA	0.0877193
3	3:20096401	20096401	T	G	intergenic	GOLPH3L, ENSA	0.0877193
3	3:20103230	20103230	G	A	UTR3	ENSA	0.0877193
3	3:20104193	20104193	T	G	UTR3	ENSA	0.0877193
3	3:20105841	20105841	G	A	downstream	ENSA	0.0877193
3	3:96048959	96048959	T	C	intergenic	DMRTA2, ELAVL4	0.0877193
3	3:96069890	96069890	A	C	intergenic	DMRTA2, ELAVL4	0.0877193
3	3:109247422	109247422	T	C	intergenic	GRIK3, MRPS15	0.0877193

Chr	Variant ID	Position (bp)	Ref	Alt	Region type	Genes	Allele frequency
3	3:109251851	109251851	T	C	intergenic	GRIK3, MRPS15	0.0877193
4	4:100949366	100949366	G	T	intronic	PTN	0.0877193
4	4:100957622	100957622	T	C	intronic	PTN	0.0877193
4	4:100959190	100959190	A	G	intronic	PTN	0.0877193
4	4:100960966	100960966	G	A	intronic	PTN	0.0877193
4	4:112096440	112096440	G	A	intergenic	EZH2, PDIA4	0.0877193
4	4:112102070	112102070	G	T	intergenic	EZH2, PDIA4	0.0877193
4	4:112105618	112105618	A	G	intergenic	EZH2, PDIA4	0.0877193
4	4:112106474	112106474	C	T	intergenic	EZH2, PDIA4	0.0892857
5	5:41753969	41753969	T	C	intronic	KIF21A	0.0877193
6	6:48308183	48308183	C	T	intergenic	STIM2, TRNAS-GGA	0.0964912
6	6:50254023	50254023	T	C	intronic	PCDH7	0.0877193
6	6:60437995	60437995	G	A	intronic	LIMCH1	0.0877193
6	6:62128591	62128591	G	A	intergenic	GRXCR1, TRNAC-ACA	0.0877193
6	6:62152514	62152514	A	G	intergenic	GRXCR1, TRNAC-ACA	0.0877193
6	6:62221849	62221849	T	G	intergenic	GRXCR1, TRNAC-ACA	0.0877193
6	6:62271501	62271501	G	A	intergenic	GRXCR1, TRNAC-ACA	0.0892857
6	6:70980137	70980137	T	C	intergenic	CLOCK, PDCL2	0.0877193
6	6:70980174	70980174	T	C	intergenic	CLOCK, PDCL2	0.0877193
6	6:70980352	70980352	C	T	intergenic	CLOCK, PDCL2	0.0877193
6	6:70980494	70980494	T	C	intergenic	CLOCK, PDCL2	0.0877193
6	6:70980687	70980687	G	A	intergenic	CLOCK, PDCL2	0.0964912
6	6:70980776	70980776	A	G	intergenic	CLOCK, PDCL2	0.0877193
6	6:70981672	70981672	C	T	intergenic	CLOCK, PDCL2	0.0877193
6	6:70982000	70982000	T	C	intergenic	CLOCK, PDCL2	0.0982143

Chr	Variant ID	Position (bp)	Ref	Alt	Region type	Genes	Allele frequency
6	6:70982176	70982176	C	T	intergenic	CLOCK, PDCL2	0.0982143
6	6:70982323	70982323	G	A	intergenic	CLOCK, PDCL2	0.0877193
6	6:70982970	70982970	A	G	intergenic	CLOCK, PDCL2	0.0877193
6	6:70984110	70984110	C	T	intergenic	CLOCK, PDCL2	0.0877193
6	6:70984139	70984139	T	G	intergenic	CLOCK, PDCL2	0.0877193
6	6:70984164	70984164	G	T	intergenic	CLOCK, PDCL2	0.0877193
6	6:70985487	70985487	C	A	intergenic	CLOCK, PDCL2	0.0964912
6	6:70987119	70987119	T	C	intergenic	CLOCK, PDCL2	0.0877193
6	6:70987197	70987197	C	T	intergenic	CLOCK, PDCL2	0.0964912
6	6:70988366	70988366	A	T	downstream	PDCL2	0.0877193
6	6:70988590	70988590	T	G	UTR3	PDCL2	0.0964912
6	6:70988604	70988604	C	T	UTR3	PDCL2	0.0964912
6	6:70988676	70988676	T	G	UTR3	PDCL2	0.0877193
6	6:70989595	70989595	A	G	intronic	PDCL2	0.0877193
6	6:70990604	70990604	G	A	intronic	PDCL2	0.0877193
6	6:70992523	70992523	T	C	intronic	PDCL2	0.0877193
6	6:70992605	70992605	G	A	intronic	PDCL2	0.0877193
6	6:70993330	70993330	T	A	intronic	PDCL2	0.0877193
6	6:70994170	70994170	G	T	intronic	PDCL2	0.0877193
6	6:70994221	70994221	G	A	intronic	PDCL2	0.0877193
6	6:70994222	70994222	C	T	intronic	PDCL2	0.0877193
6	6:70994519	70994519	C	T	intronic	PDCL2	0.0877193
6	6:70994527	70994527	T	C	intronic	PDCL2	0.0877193
6	6:70994777	70994777	A	G	intronic	PDCL2	0.0877193
6	6:70994815	70994815	G	A	intronic	PDCL2	0.0877193

Chr	Variant ID	Position (bp)	Ref	Alt	Region type	Genes	Allele frequency
6	6:70995434	70995434	A	C	intronic	PDCL2	0.0877193
6	6:70995844	70995844	A	G	intronic	PDCL2	0.0877193
6	6:70995876	70995876	G	A	intronic	PDCL2	0.0877193
6	6:70996189	70996189	G	A	intronic	PDCL2	0.105263
6	6:76343901	76343901	G	C	intergenic	NONE, TRNAC-GCA	0.0877193
6	6:97859325	97859325	A	C	intronic	COPS4	0.0877193
6	6:97889818	97889818	C	T	intronic	PLAC8	0.0877193
7	7:99221350	99221350	G	A	intergenic	CHD1, FAM174A	0.0964912
7	7:104164032	104164032	G	A	intergenic	LOC112447533, NONE	0.105263
8	8:109022323	109022323	G	A	intergenic	BRINP1, CDK5RAP2	0.0877193
10	10:59233183	59233183	A	G	intergenic	CYP19A1, TNFAIP8L3	0.122807
14	14:32382887	32382887	G	A	intronic	C14H8orf34	0.0964912
14	14:32382888	32382888	G	C	intronic	C14H8orf34	0.0964912
15	15:19790729	19790729	T	C	intergenic	C15H11orf87, TRNAR-CCU	0.122807
15	15:19790737	19790737	T	C	intergenic	C15H11orf87, TRNAR-CCU	0.0964912
15	15:25613931	25613931	A	G	intergenic	NXPE2, CADM1	0.0877193
15	15:50899116	50899116	A	G	intergenic	LOC788363, TRNAG-CCC	0.0877193
15	15:51630012	51630012	G	T	intronic	RNF121	0.0964912
15	15:51630018	51630018	G	T	intronic	RNF121	0.0964912
15	15:51703924	51703924	C	A	intergenic	NUMA1, LRRC51	0.0964912
15	15:58987849	58987849	A	G	intronic	METTL15	0.0877193
15	15:59046599	59046599	C	T	intronic	METTL15	0.0877193
15	15:59077810	59077810	A	G	downstream	METTL15	0.0877193
16	16:25966976	25966976	A	T	intergenic	DUSP10, TRNAT-UGU	0.0877193
16	16:25967624	25967624	A	G	intergenic	DUSP10, TRNAT-UGU	0.0877193

Chr	Variant ID	Position (bp)	Ref	Alt	Region type	Genes	Allele frequency
16	16:25967632	25967632	G	A	intergenic	DUSP10, TRNAT-UGU	0.0877193
16	16:25971304	25971304	C	T	intergenic	DUSP10, TRNAT-UGU	0.0877193
16	16:25971690	25971690	G	A	intergenic	DUSP10, TRNAT-UGU	0.0877193
16	16:25971723	25971723	T	G	intergenic	DUSP10, TRNAT-UGU	0.0877193
16	16:25972014	25972014	G	A	intergenic	DUSP10, TRNAT-UGU	0.0877193
16	16:25973431	25973431	A	G	intergenic	DUSP10, TRNAT-UGU	0.0877193
16	16:25973729	25973729	G	A	intergenic	DUSP10, TRNAT-UGU	0.0877193
16	16:25975114	25975114	G	A	intergenic	DUSP10, TRNAT-UGU	0.0877193
16	16:25975166	25975166	G	A	intergenic	DUSP10, TRNAT-UGU	0.0877193
16	16:25976447	25976447	C	T	intergenic	DUSP10, TRNAT-UGU	0.0877193
16	16:25977875	25977875	C	T	intergenic	DUSP10, TRNAT-UGU	0.0877193
16	16:25989143	25989143	C	T	intergenic	DUSP10, TRNAT-UGU	0.0877193
16	16:59590393	59590393	T	C	intronic	RASAL2	0.0964912
17	17:3173060	3173060	G	A	intronic	DCHS2	0.0877193
17	17:14459741	14459741	G	T	intronic	GAB1	0.0877193
17	17:16163383	16163383	G	A	intergenic	IL15, ZNF330	0.0877193
17	17:28778920	28778920	G	C	intronic	SCLT1	0.0877193
17	17:28803567	28803567	C	T	intronic	SCLT1	0.0877193
18	18:6033493	6033493	T	C	intronic	WWOX	0.0877193
18	18:6044938	6044938	G	C	intronic	WWOX	0.0877193
19	19:9573214	9573214	T	C	intronic	TEX14	0.0877193
19	19:9580708	9580708	G	C	intronic	TEX14	0.0877193
19	19:9584425	9584425	C	T	intronic	TEX14	0.0877193
19	19:9586853	9586853	G	A	intronic	TEX14	0.0877193
19	19:9590209	9590209	C	T	intronic	TEX14	0.0877193

Chr	Variant ID	Position (bp)	Ref	Alt	Region type	Genes	Allele frequency
19	19:9592593	9592593	T	C	intronic	TEX14	0.0877193
19	19:9593850	9593850	G	A	intronic	TEX14	0.0877193
19	19:9595204	9595204	A	C	intronic	TEX14	0.0877193
19	19:9610970	9610970	A	T	intronic	TEX14	0.0877193
19	19:9616703	9616703	C	G	intronic	TEX14	0.0877193
19	19:9622770	9622770	A	G	intronic	TEX14	0.0877193
19	19:9625318	9625318	A	G	intronic	TEX14	0.0877193
19	19:9627686	9627686	G	A	intronic	TEX14	0.0877193
19	19:9628869	9628869	A	G	intronic	TEX14	0.0964912
19	19:9641928	9641928	A	C	intronic	TEX14	0.0877193
19	19:9644729	9644729	A	G	intronic	TEX14	0.0877193
19	19:9650457	9650457	G	A	intronic	TEX14	0.0964912
19	19:9653551	9653551	G	A	intronic	TEX14	0.0964912
20	20:4419150	4419150	T	A	intronic	NEURL1B	0.0877193
20	20:57716533	57716533	C	T	intergenic	FBXL7, ANKH	0.0943396
20	20:57716534	57716534	A	G	intergenic	FBXL7, ANKH	0.0943396
20	20:60790411	60790411	G	A	intergenic	DNAH5, CTNND2	0.0877193
21	21:25303635	25303635	T	C	intronic	RASGRF1	0.0877193
21	21:54401385	54401385	T	C	intergenic	FSCB, C21H14orf28	0.0877193
22	22:17713072	17713072	C	T	intergenic	RAD18, OXTR	0.0877193
22	22:17713077	17713077	G	A	intergenic	RAD18, OXTR	0.0877193
22	22:17713080	17713080	A	G	intergenic	RAD18, OXTR	0.0877193
22	22:31869929	31869929	A	G	intergenic	MITF, FRMD4B	0.0964912
23	23:17690492	17690492	C	T	intronic	CAPN11	0.0877193
23	23:27872738	27872738	G	C	exonic	BOLA	0.0877193

Chr	Variant ID	Position (bp)	Ref	Alt	Region type	Genes	Allele frequency
23	23:36338915	36338915	G	A	intergenic	MIR2284C, SOX4	0.0982143
23	23:36338918	36338918	A	T	intergenic	MIR2284C, SOX4	0.0982143
23	23:36889007	36889007	A	G	intronic	CDKAL1	0.0877193
26	26:48889214	48889214	T	C	intronic	MGMT	0.0877193
26	26:48891758	48891758	C	T	intronic	MGMT	0.0877193
26	26:48892917	48892917	C	G	intronic	MGMT	0.0877193
28	28:4746702	4746702	A	G	intronic	DISC1	0.0964912
29	29:23720365	23720365	G	A	intronic	NELL1	0.0877193
29	29:23720373	23720373	G	C	intronic	NELL1	0.0877193
29	29:23720376	23720376	A	G	intronic	NELL1	0.0877193
29	29:27122151	27122151	G	C	intergenic	LOC781828, LOC100850308	0.0877193
29	29:27127685	27127685	A	G	intergenic	LOC781828, LOC100850308	0.0877193
